# Zhili decoction ameliorates ulcerative colitis by modulating gut microbiota and related metabolite, and inhibiting the TLR4/NF-κB/NLRP3 pathway

**DOI:** 10.3389/fphar.2024.1481273

**Published:** 2024-12-23

**Authors:** Tianying Tan, Qin Chen, Ping Chen, Shuangshuang Li, Wenting Hu, Tao Yang, Yingtian Jia

**Affiliations:** ^1^ College of Clinical Medicine, Guizhou University of Traditional Chinese Medicine, Guiyang, China; ^2^ Department of Anorectal, Kunming Municipal Hospital of Traditional Chinese Medicine, The Third Affiliated Hospital of Yunnan University of Chinese Medicine, Kunming, China; ^3^ Colorectal and Anal Surgery, Chongqing Jiangbei District Hospital of Traditional Chinese Medicine, Chongqing, China; ^4^ Colorectal and Anal Surgery, The Affiliated Traditional Chinese Medicine Hospital of Southwest Medical University, Luzhou, China; ^5^ Colorectal and Anal Surgery, The First Affiliated Hospital of Guizhou University of Traditional Chinese Medicine, Guiyang, China

**Keywords:** ulcerative colitis, Zhili decoction, gut microbiome, metabolism, TLR4/NF-κB/NLRP3 pathway

## Abstract

**Ethnopharmacological Importance:**

Zhili decoction (ZLD) is a traditional Chinese medicine prescription for ulcerative colitis (UC). However, the mechanism by which ZLD exerts its therapeutic effects in the context of UC remains unclear.

**Aim of study:**

The aim of this study was to investigate the effects of ZLD on the gut microbiota and related fecal metabolite levels using a mouse model of UC. In addition, we examined the underlying molecular mechanisms responsible for these effects.

**Materials and methods:**

The major components of ZLD were detected by ultra-performance liquid chromatography-mass spectrometry (UPLC-MS). An integrated approach employing 16S rRNA and fecal metabolomics was employed to assess the potential impacts of ZLD on gut flora abundance and diversity, fecal metabolite levels, and various metabolic pathways. To further investigate the potential mechanisms of ZLD in treating UC, the expression of genes and proteins were examined by RT-qPCR, immunohistochemical staining and Western blotting.

**Results:**

ZLD markedly alleviated symptoms and inflammatory injury in mice with UC. DSS induced notable alterations in the gut microbiome, and ZLD enhanced gut microbial diversity in UC mice by augmenting the abundance of Bacteroidota, Christensenella, *Lactobacillus*, etc., while reducing the *Firmicutes*/*Bacteroidota* ratio. ZLD treatment significantly modified the metabolic profile of mice with UC. It significantly suppressed the arachidonic acid metabolic pathway and promoted the butyrate acid metabolic pathway. ZLD reduced inflammatory factors and inhibited TLR4/NF-κB/NLRP3 pathway expression. In addition, correlation analysis demonstrated a close relationship between gut microbes, fecal metabolites, and inflammatory factors.

**Conclusion:**

ZLD alleviated UC by regulating gut flora, modulating related metabolite levels, and inhibiting TLR4/NF-κB/NLRP3 pathway.

## 1 Introduction

Ulcerative colitis (UC) is persistent inflammation that extends from the mucosal end of the rectum toward the colonic end, often progressing over time (Eisenstein, 2018; Ng et al., 2017). Symptoms include bloody diarrhea, pus, and abdominal pain. The global incidence of UC is on the rise, particularly among younger individuals, possibly due to lifestyle and dietary changes, as well as environmental factors (Molodecky et al., 2012). This increasing prevalence makes UC a major global public health concern, impacting the wellbeing of individuals worldwide. While current treatment options like anti-inflammatory medications and anti-tumor necrosis factor (TNF) targeted therapies can be used to manage moderate to severe symptoms (Nakase et al., 2021; Salice et al., 2019), they often induce severe adverse effects, including liver and kidney impairment or an increased risk of infections (Ford and Peyrin-Biroulet, 2013; Luthra et al., 2015). Thus, advancing safe and economical therapeutic approaches for UC remains a critical priority in medical research.

The intestinal microbiota constitutes a pivotal element of the intestinal barrier, influencing various physiological functions. It facilitates nutrient absorption and metabolism, and regulates the immune system (Round and Mazmanian, 2009). Beneficial microbiota actively counteract potential pathogens, preserving mucosal integrity and immune homeostasis (Honda and Littman, 2016; Mu et al., 2017). Nevertheless, research indicates a close association between intestinal microbiota and UC development (Frank et al., 2007). Patients with UC exhibit reduced microbial diversity, with a decline in beneficial species and an increase in harmful microorganisms (Ni et al., 2017). This imbalance affects related-metabolite production, decreasing the levels of short-chain fatty acids (SCFAs) (Parada Venegas et al., 2019). SCFAs, encompassing acetic, propionic, and butyrate acids, represent metabolic byproducts of beneficial bacteria generated through the intestinal fermentation of dietary fiber. They exhibit anti-inflammatory and antioxidant effects by activating G protein-coupled receptors on colonic epithelial cells, inhibiting the level of pro-inflammatory factors, and controlling immune responses. This promotes the intestinal mucosal barrier to repair and maintain its integrity (Morrison and Preston, 2016). Thus, disruptions in microbial communities alter metabolite levels, affecting host metabolism and triggering UC(Zhou et al., 2020). Targeting gut flora alterations may provide an avenue for UC management.

Zhili Decoction (ZLD) is a traditional compound formula documented in the Color Atlas of Hmong Medicines in Traditional Chinese Medicine, comprising *Acalypha australis* L. (AAL) (known as Tie Xian Cai in Chinese) and *Phellodendron chinense* var. *glabriusculum C.K. Schneid.* (Huang Pi Shu or Chuan Huang Bai in Chinese), is a traditional remedy known for treating diarrhea and dysentery. AAL exhibits anti-inflammatory properties and promotes apoptosis (Kim et al., 2020). It has been used to treat dysentery and diarrhea. Additionally, AAL reduces the production of interleukin (IL)-6 and tumor necrosis factor (TNF)-α by inhibiting the activation of NF-κB (Chen et al., 2023). In contrast, Phellodendron chinense var. glabriusculum C.K. Schneid. has anti-inflammatory, analgesic, and uric acid-lowering properties, and helps regulate intestinal flora (Chan et al., 2008; Chen et al., 2010). Both herbal components demonstrate therapeutic effects on ulcerative colitis (UC) by reducing nitric oxide (NO), IL-1, and IL-1β levels (Park et al., 2007). However, its underlying mechanism of action against UC remains unclear. This study aims to explore the efficacy and potential mechanisms of ZLD in UC.

## 2 Materials and methods

### 2.1 Reagents

The details regarding the reagents utilized in this investigation can be found in Supplementary Table S1.

### 2.2 Preparation of ZLD

The fresh bark of *Phellodendron chinense* var. *glabriusculum C.K.Schneid*. and *Acalypha australis* L. (Dechangxiang, Guizhou) was crushed and blended in a 1:1 ratio. Subsequently, 1000 g of ZLD was soaked in 10 L of water for 1 h, refluxed at 100°C for 2 h, filtered and extracted with 8000 mL of water at the same temperature for another 1.5 h. The resulting filtrates were combined, condensed, and subjected to vacuum freeze-drying to yield a powdered extract. Finally, a 58.2g ZLD was obtained for the follow-up experiment.

### 2.3 ZLD chemical analysis

0.5g ZLD powder was dissolved with 10 mL distilled water and extracted with 50% methanol. After vibration and centrifugation at 12,000 rpm for 30 min, the supernatant was transferred and evaporated to dryness at a temperature under vacuum. The residue was dissolved in 1 mL of ddH2O and centrifugated at 12,000 rpm for 15 min twice prior to analysis. The standard substances were dissolved in methanol. The chemical analyses were performed using Thermo Fisher Q Exactive coupled with a Vanquish UPLC system. The mobile phase in positive mode included H2O with 0.1% FA (A) and methanol (B). The gradient conditions included: 0–2 min at 10% B; 2–3 min at 10%–35% B; 3–10 min at 35%–45% B; 10–11 min at 45%–90% B; 11–12 min at 90% B; and 12–12.5 min at 90%–10% B. The mobile phase also consisted of 0.1% FA (C) and methanol (D) under negative mode. The negative mode gradient conditions are: 0–2 min at 10% D; 2–3 min at 10%–35% D; 3–5 min at 35%–90% D; 5–7 min at 90%–95% D; 7–9 min at 95% D; 9–11 min at 95%–10% D. The experimental conditions included a flow rate of 200 μL/min and a constant temperature of 40°C. The mass conditions were: spray voltage: +3.4KV/-3 KV; sheath gas pressure: 40 arb; aux gas pressure: 10 arb; capillary temp: 300°C; heater temp: 320°C.

### 2.4 Animals and ethical approval

Eight-week-old specific pathogen-free (SPF) C57BL/6J mice, weighing 20 ± 2 g, were obtained from Beijing Huafukang Animal Breeding Center (License No. SCXK-[jing]-2019–0008). Mice were adapted and fed for 1 week, and experimental phase keeping was performed under standard laboratory conditions. The experimental procedures received approval from Guizhou University of Traditional Chinese Medicine Animal Research Institute (approval number 20230047).

### 2.5 UC model and drug administration

UC induction with dextran sulfate sodium (DSS) was performed as previously reported. Mice were kept in a controlled space under SPF conditions and received sterile diet and water. Experimental mice were divided randomly into control, DSS, sulfasalazine (SASP), ZLD low-dose (ZLD-L), and ZLD high-dose (ZLD-H) groups (n = 15 per group). UC was induced in groups DSS, SASP, ZLD-L, and ZLD-H by administering 3% DSS (w/v) over a period of 7 consecutive days. Mice in the control group were provided with deionized drinking water for a period of 14 days. Following UC induction, the reference group mice were administered 125 mg/kg/d SASP. Mice in the ZLD-L and ZLD-H groups were fed 291.25 mg/kg/d and 873.75 mg/kg/d ZLD, respectively. SASP and ZLD were administered orally once daily for 7 days. All treated mice were euthanized on the 14th day.

The severity of the disease was assessed daily basis by monitoring body weight, dietary intake, fecal occult blood, and consistency of stool. The Disease Activity Index (DAI) was computed based on established criteria (Li et al., 2021) (Supplementary Table S2). DAI scores were calculated by summing the weight change, defecation, and occult blood scores, and then dividing it by three. On day 13, fresh feces were harvested from each mouse and frozen at −80°C in sterile EP tubes. The following day, colon tissues were obtained also stored at −80°C.

### 2.6 Histopathologic analysis

Colon tissues from the mice were fixed in a 4% paraformaldehyde solution, then embedded in paraffin and subsequently sectioned at a thickness of 5 μM. The sections were processed for hematoxylin and eosin (HE) staining, followed by visualization and photography under a light microscope (Nikon Fi3, Japan). Pathological scoring of colonic injury was conducted using the Geboes score (Supplementary Table S3) (Shi et al., 2017).

### 2.7 Staining by transmission electron microscopy (TEM)

Colon tissues (<1 mm³) were fixed in 2.5% glutaraldehyde and phosphate buffer solution. Following fixation, gradual dehydration was performed using progressive ethanol concentrations. After embedding and solidification, sections of 70 nm were cut using an ultramicrotome (Leica UC7). These sections were stained with electron microscopy dyes to enhance contrast for cellular organelles and structures. Subsequently, the samples were observed for their ultrastructure using 80 kV transmission electron microscopy (HT7800/HT7700), and images were captured.

### 2.8 ELISA assay

Add 10 mg of tissue to 500 μL of extract (containing protease inhibitor and Ripa lysate) and homogenize using a homogenizer. The homogenate was subjected to centrifugation at a speed of 12,000 revolutions per minute for a duration of 10 min at a temperature of 4°C. Following this, the supernatant was carefully extracted for use in the ELISA assay. Pro-inflammatory cytokine levels, including TNF-α, IL-6, IL-1β, and IL-18, were assessed using an ELISA kit (Sevier, China) as per the manufacturer’s guidelines.

Fecal samples were selected and subjected to three freeze-thaw cycles at −20°C. They were then filtered and extracted using a butanol: methanol: water (5:25:70, v/v/v) solution. The specific metabolites were quantified following the instructions provided with the ELISA kits.

### 2.9 Immunohistochemical (IHC) assay

Paraffin sections of colon tissue were stained using rabbit polyclonal antibodies against Toll-like receptor 4 (TLR4, dilution 1:400) and NLR family pyrin domain containing 3 (NLRP3, dilution 1:50), along with polyclonal anti-rat IgG (1:200) antibodies. Section images were taken with NIS Elements Imaging Software Version 4.0.

### 2.10 Real-time quantitative (RT-q) PCR analysis

Total RNA was extracted using the Tissue RNA Purification kit (EZBioscience, China), following the manufacturer’s guidelines. Quantify RNA using the kit (refer to Supplementary Table S1 for details). RT-qPCR analysis was conducted using the CFX96 Real-Time System (BIO-RAD, United States). Supplementary Table S4 contains the primer sequences used in this study.

### 2.11 Western blotting (WB) analysis

FastPrep-24TM5G (MP Biomedicals, United States) for extraction of total proteins from PBS-washed colon tissues. The specific experimental procedures of the Western blot have been cited in previous studies (Yang et al., 2024). Used the following antibodies at the indicated dilutions: β-actin (dilution 1:100,000), NF-κB (dilution 1:1,000), Phospho-inhibitor of NF-κB alpha (p-IKBα, dilution 1:1,000), cleaved caspase-1 (dilution 1:3,000), apoptosis-associated speck-like protein containing a CARD (ASC; dilution 1:1,000), NLRP3 (dilution 1:500), and HRP-conjugated (dilution 1:10,000). Bands were visualized and quantified utilizing the ChemiDocTM Imaging System (BIO-RAD, United States) and Image Lab software.

### 2.12 16S rRNA sequencing analysis for intestinal flora

Genomic DNA of feces collected from control, DSS, ZLD-L, and ZLD-H groups was extracted using the DNA kit (Omega Bio-Tek, China) according to the instructions. The V3-V4 hypervariable regions of the bacterial 16S rRNA gene were amplified from genomic DNA using specific primers (338F 5′-ACT​CCT​ACG​GGA​GGC​AGC​A-3′ and 806R 5′-GGACTACHVGGGTWTCTAAT-3′). Samples were individually labeled with unique barcodes during PCR amplification, utilizing Tks Gflex DNA Polymerase. The quantity and quality of the amplified products were assessed with the gel electrophoresis. Following this, a purification of the PCR product was conducted using VAHTSTM DNA Clean Beads and AMPure XP Beads, respectively. The purified PCR products were characterized with the Quant-iT PicoGreen dsDNA Assay Kit. Libraries were constructed and sequenced using the MiSeq Reagent Kit v3 (Illumina, United States). Raw data were generated in FASTQ format. Microbiome biological information was analyzed using QIIME2 to obtain Operational Taxonomic Units (OTU). The index of Chao1, Shannon, and Simpson, as well as principal coordinates analysis (PCoA) and non-metric multidimensional scaling (NMDS) plots, were generated using R software and QIIME2 software. To detect taxonomic units that differ significantly between groups, we used the Least Discriminant Analysis Effect Size (LEfSe) method.

### 2.13 Fecal metabolomic data analysis

Referring to previous studies described (Lu et al., 2021), to implement the operation, metabolite extraction involved adding 50 mg of fecal sample to 400 μL of extraction solution (methanol: water = 4:1) containing 0.02 mg/mL of the internal standard (L-2-chlorophenylalanine) in an EP tube. The supernatant obtained after sample grinding was pipetted into an analytical vial. The UPLC-Q Exactive system (Thermo Fisher Scientific) with an HSS T3 column (100 mm × 2.1 mm, 1.8 μm) and Q Exactive mass spectrometry were used for detection. The mobile phases were composed of a solution of 0.1% formic acid in water and acetonitrile (95:5, v/v) (referred to as solvent A), as well as a solution of 0.1% formic acid in acetonitrile, isopropanol, and water (47.5:47.5:5, v/v) (referred to as solvent B) (Zhang et al., 2023). The automatic injector was set to 4°C and programmed to dispense a volume of 3 μL. Information Dependent Acquisition (IDA) controlled by Xcalibur software (Thermo-Fisher) was used to collect mass spectrometry data. The mass spectra were continuously assessed in positive and negative status for further analysis in the subsequent stage.

### 2.14 Statistical analysis

Data were subjected to analysis using GraphPad Prism 9.0 software. The student’s test and one-way ANOVA were used, and all data were expressed as mean ± standard error of the mean (SEM). The co-occurrence among intestinal flora and metabolites was calculated based on the relative abundance by Spearman’s rank correlation coefficient (*P*< 0.05) using R package Hmisc. Visualization of results was achieved using R 4.03. Differences were considered statistically significant for *P*-values < 0.05.

## 3 Results

### 3.1 Chemical analysis of ZLD extract

**FIGURE 1:**
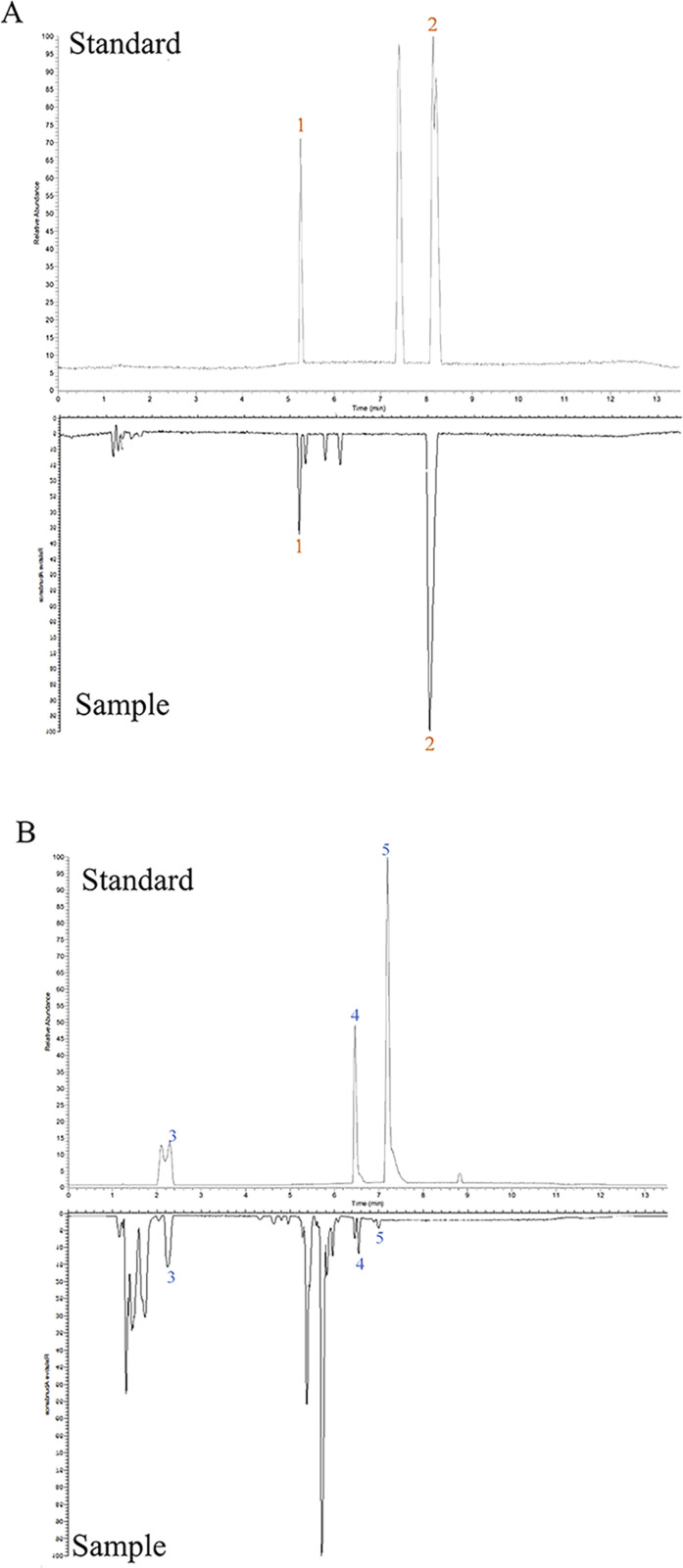
Chemical analysis of ZLD extract under positive **(A)** and negative **(B)** modes. 1, phellodendrine; 2, berberine; 3, gallic acid; 4, quercetin; 5, galangin.

The chemical analysis showed that 108 compounds were contained in ZLD (Supplementary Table S5). Among them, hellodendrine and berberine were identified under the positive mode, according to the standard substances (Figure 1A). The three chemicals in the ZLD extract were identified as gallic acid, quercetin, and galanin (Figure 1B).

### 3.2 The evolution of DSS-induced UC in mice

**FIGURE 2:**
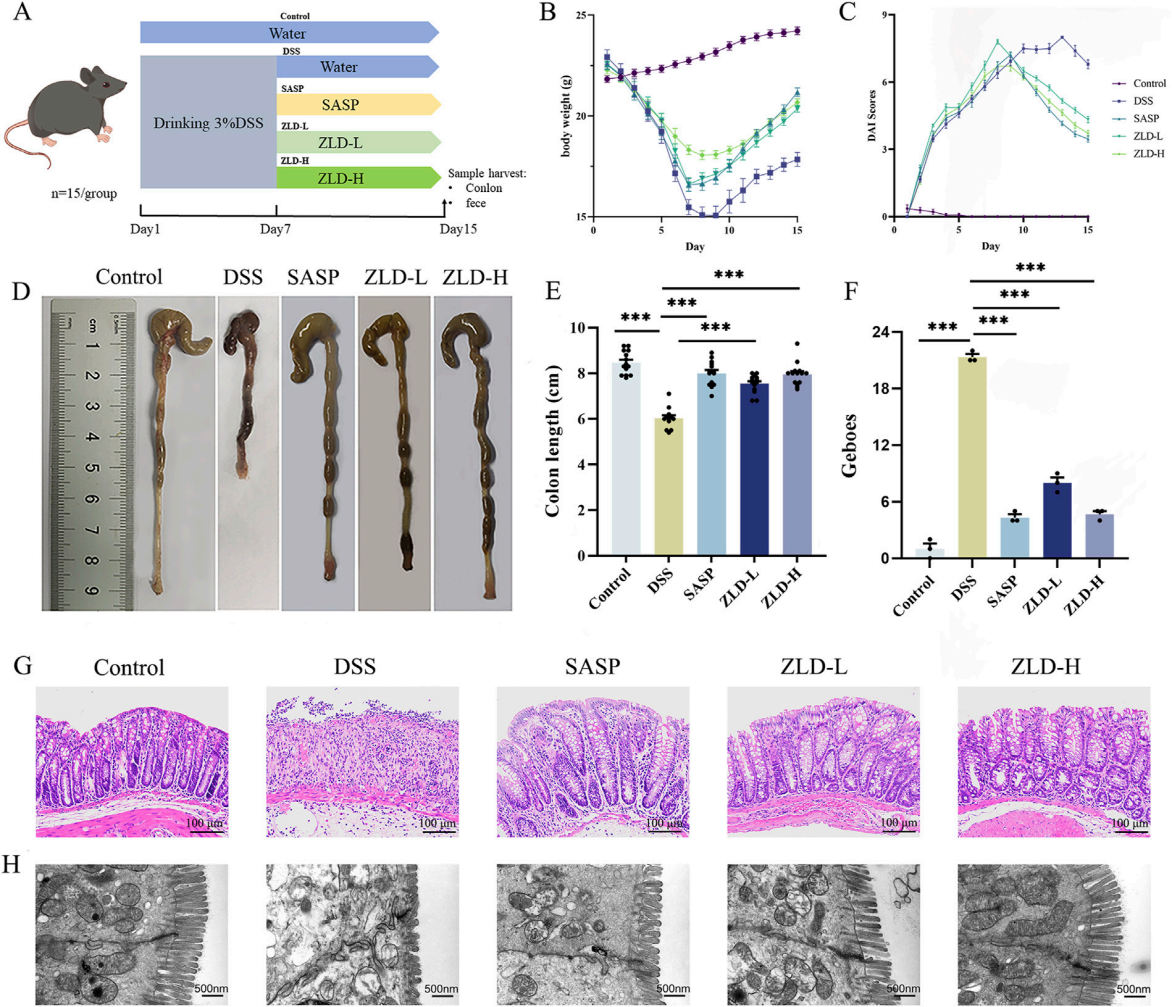
**(A)** Schematics of the experimental design. **(B, C)** Changes in body weight and DAI for mice. **(D, E)** Colon length measurements and comparisons across different groups. **(F, G)** Histopathologic picture of mouse colon and histological scores of colonic tissues (magnification × 200). **(H)** Representative images of colon tissues obtained using transmission electron microscopy (scale bars = 500 nm). ZLD-L: low dose ZLD; ZLD-H: high dose ZLD; all groups: n = 15; *p < 0.05, **p < 0.01, ***p < 0.001, ^ns^ *p* > 0.05.

Protocol design is illustrated in Figure 2A. In contrast to the control mice, the DSS group exhibited a reduction in body weight (Figure 2B), elevated DAI score (Figure 2C), and shortened colon length (Figures 2D, E). Histopathological examination revealed extensive inflammatory cell infiltration and ulceration in the colon mucosa of the DSS group compared with the other experimental groups, with significantly higher Geboes values (Figures 2F, G). Treatment with SASP, ZLD-L, and ZLD-H significantly ameliorated the pathological alterations of the colon tissue and reduced the Geboes values (Supplementary Table S3). In addition, TEM results showed that the intestinal barrier and tight junctions were disrupted in the DSS group (Figure 2H). Nevertheless, the ZLD-L and ZLD-H groups exhibited significantly improved intestinal barrier structures as compared to the model group, while the SASP group did not show a significant improvement.

### 3.3 ZLD altered the diversity and composition of the gut microbiome

**FIGURE 3:**
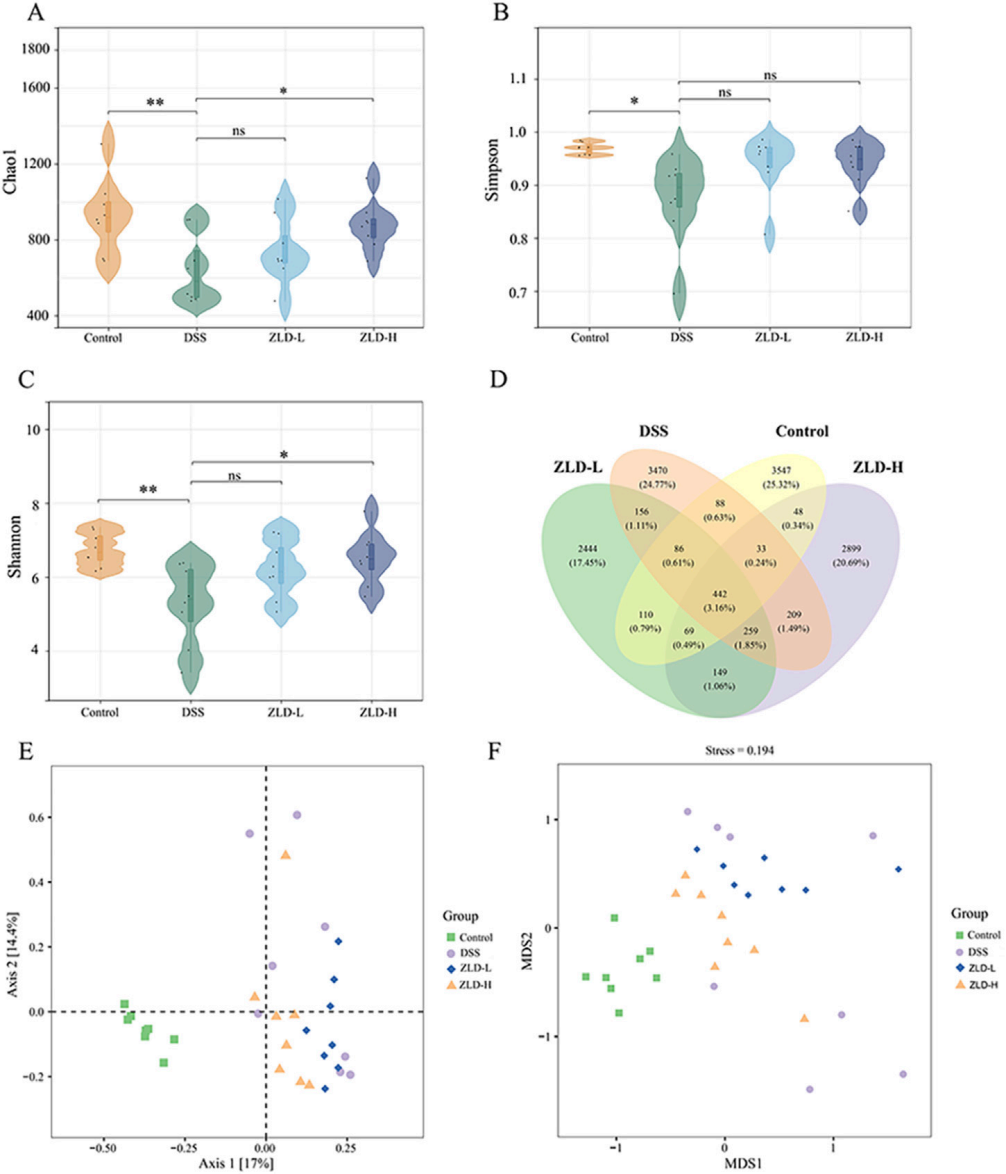
Gut microbiome diversity (n = 8 per group). α diversity compared by Chao1 **(A)** Simpson **(B)** and Shannon **(C)** index. **(D)** Venn plot of the out numbers in each group; **(E)** PCoA plots; **(F)** NMDS plots; ^*^ *p* < 0.05, ^*^ *p* < 0.01, ^***^ *p* < 0.001, ^ns^ *p* > 0.05.

Mouse fecal samples were subjected to high-throughput 16S rRNA sequencing in order to evaluate the impact of ZLD on gut flora. We utilized sparse curves, as well as α and β diversity indices, to assess the microbial species diversity present in the samples. These curves were relatively flat for each group (Supplementary Figure S1). A Venn diagram unveiled a combined total of 442 OTUs across the four groups, as illustrated in Figure 3D. To assess alpha diversity in the gut microbiota, Chao1, Simpson, and Shannon indices were utilized at the OTU level. Compared to other groups, the DSS group exhibited reduced.

Chao1, Simpson, and Shannon indices, indicating decreased microbiota richness and diversity (Figures 3A–C). Treatment with ZLD-L and ZLD-H significantly raised Chao1, Simpson, and Shannon indices values (Chao1 and Shannon: *P*< 0.05) versus the DSS group. However, differences in those three indices for the ZLD-L group and in the Simpson index for the ZLD-H were not significant. PCoA and NMDS were conducted to evaluate the β diversity across groups. Microbial community analysis using PCoA and NMDS revealed distinct clustering patterns between groups. The DSS group displayed a broader distribution than the control group, while the ZLD-L and ZLD-H groups demonstrated distributions similar to the control group (Figures 3E, F). Thus, ZLD could comprehensively elevate the α and β diversity of microbiota in mice with UC.

**FIGURE 4:**
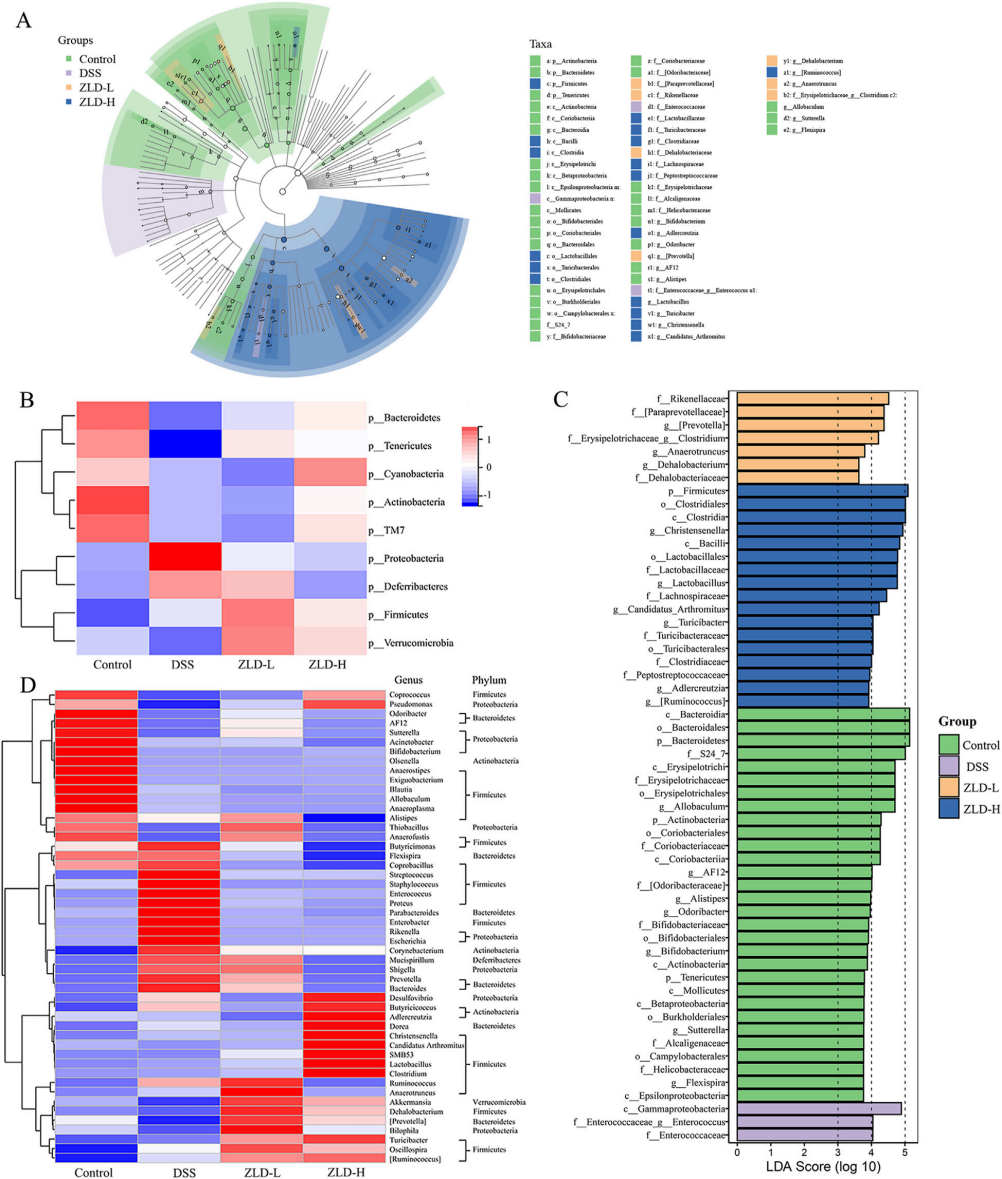
Gut microbiome composition (n = 8 per group). **(A)** Cladogramrepresenting the results of LEfSe analysis. **(B)** Heatmap of the different fecal microbiota in each group at the phylum level. Different colors indicate different metabolite expressions. **(C)** LDA scores; **(D)** The heatmap illustrates the 50 distinct genera of fecal microbiota. Different colors indicate different metabolite levels.

The gut microbiota in the DSS group was predominantly predominantly composed of the phylum level *Bacteroidota*, *Firmicutes*, and *Proteobacteria* (see Supplementary Figure S2). The relative abundance of *Bacteroidota* decreased, while that of *Firmicutes*, *Proteobacteria* increased in DSS group (Figure 4B). Additionally, the DSS group exhibited a higher *Firmicutes*/*Bacteroidota* (F/B) ratio was elevated in the DSS group than in the control group (Supplementary Figure S3). *Bacteroidota*, *Firmicutes*, *Proteobacteria,* and *Verrucomicrobia* were the main components of the intestinal flora in the ZLD-L and ZLD-H groups (Supplementary Figure S2).

Compared to the DSS group, the relative abundance of *Firmicutes* and *Bacteroidetes* increased in the ZLD-L and ZLD-H groups (Figure 4B). The F/B ratio decreased in the ZLD-H group and increased in the ZLD-L group compared to the DSS group (Supplementary Figure S3). However, neither difference was statistically significant. Additionally, ZLD treatment elevated the abundance of *Verrucomicrobia* and depressed *Proteobacteria*. The taxonomic composition by group at various levels is illustrated in Supplementary Figures S4–7 to provide further elucidation.

*Lactobacillus*, *Akkermansia*, and *Prevotella* were the predominant genera in all four groups, as illustrated in Figure 4A. LEfSe analysis was conducted to determine distinctive bacteria across the groups. In contrast to the DSS group, at the genus level, the ZLD-H exhibited an enhanced relative abundance of *Coprococcus*, [*Prevotella*], *Clostridium*, *Christensenella*, *Lactobacillus*, *Candidatus_Arthromitus*, *Turicibacter*, and degraded relative abundance of 13 other microorganisms, including *Prevotella*, *Enterococcus, Bacteroides*, *Parabacteroides*, *Ruminococcus*, *Enterococcus*, *Streptococcus*, *Staphylococcus*, *Proteus*, *Enterobacte*r, *Shigella*, *Rikenella*, *Escherichia*, and *Mucispirillum*. The relative abundance of *Turicibacter* and *Anaerofustis* was elevated, yet that of *Streptococcus*, *Staphylococcus*, *Enterococcus*, *Proteus*, *Enterobacter*, *Parabacteroides*, *Rikenella*, *Escherichia*, *Desulfovibrio*, and *Butyricicoccus* was reduced for the ZLD-L group than for the DSS group. The findings suggest that ZLD treatment induces changes in the composition and diversity of gut microbiota, as depicted in Figure 4D.

We identified 50 critical microbial species with linear discriminant analysis (LDA) scores >4 (Figure 4C). 21 key differential gut microbes species were identified utilizing LDA >4 as a criterion, with 7 signature differential gut microbes at the genus level: [*Prevotella*], *Clostridium*, *Christensenella*, *Lactobacillus*, *Candidatus_Arthromitus*, *Turicibacter*, *Enterococcus*.

### 3.4 The impact of ZLD on metabolites

**FIGURE 5:**
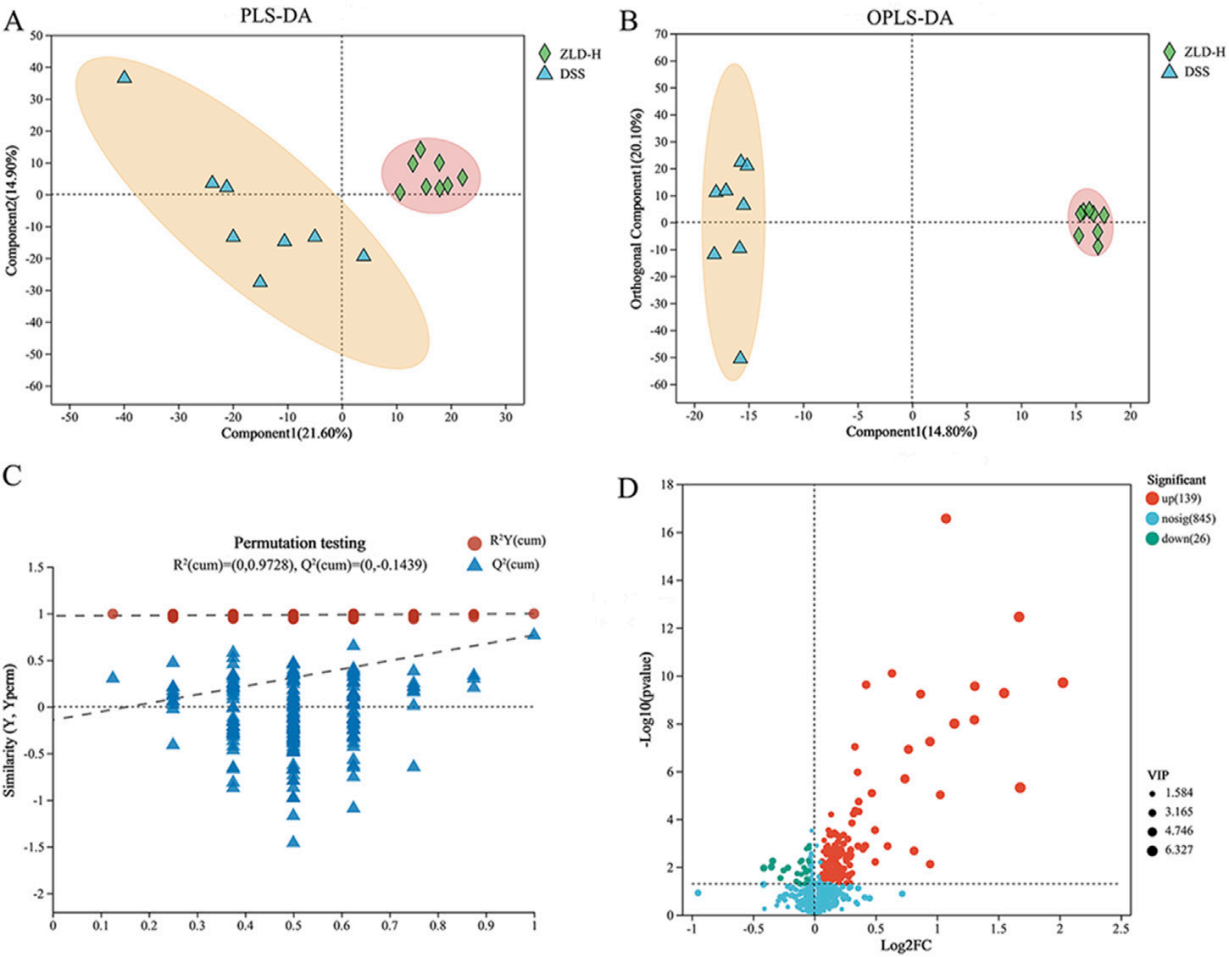
Metabolite differences between the dextran sulfate sodium and Zhili decoction high-dose treated groups in a positive model. **(A)** PLS-DA; **(B)** OPLS-DA; **(C)** OPLS-DA permutation test; **(D)** Volcano plots.

**FIGURE 6:**
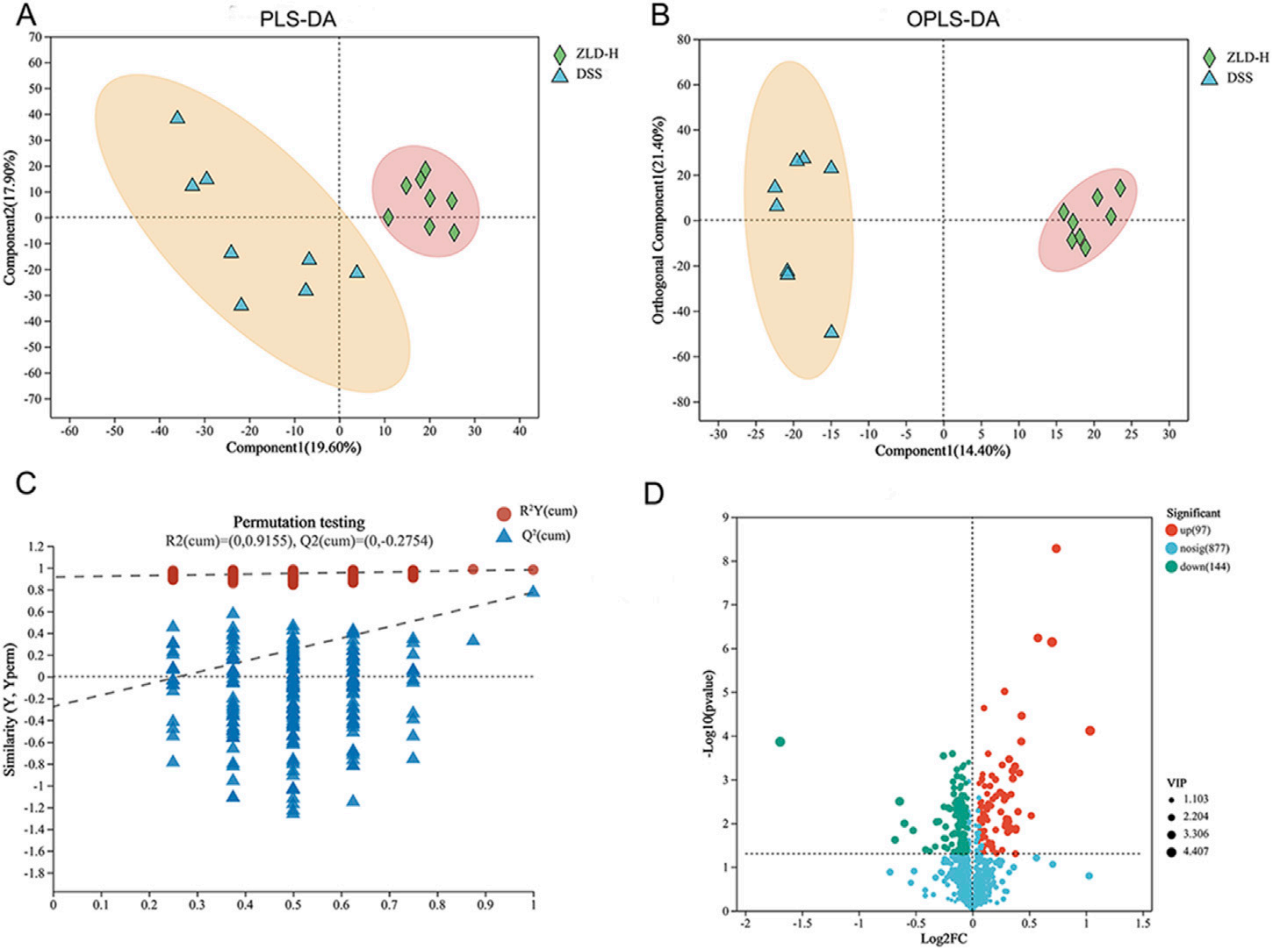
Analysis of metabolites showed differences between DSS and high-dose ZLD groups in the negative mode. **(A)** PLS-DA; **(B)** OPLS-DA; **(C)** OPLS-DA permutation test. **(D)** Volcano plots.

Our previous results indicated that, among the tested compounds, ZLD-H treatment was the most effective in managing UC. Furthermore, it significantly promoted specific beneficial flora. Subsequently, we analyzed the effect of ZLD-H on fecal metabolism using liquid chromatography-tandem mass spectrometry. Mice metabolic profiling carried out using Partial Least Squares Discriminant Analysis (PLS-DA) and Orthogonal Partial Least Squares Discriminant Analysis (OPLS-DA) (Figures 5, 6), revealed significant differences in metabolite profiles between the DSS and ZLD-H groups (Supplementary Figures S8, S9), with samples in the ZLD-H group resembling those from the control group. The DSS group formed distinct metabolic clusters compared to the ZLD-H group (cationic model: R^2^Y = 0.9728, Q^2^ = −0.1439; anionic model: R^2^Y = 0.91555, Q^2^ = −0.2754).

**FIGURE 7:**
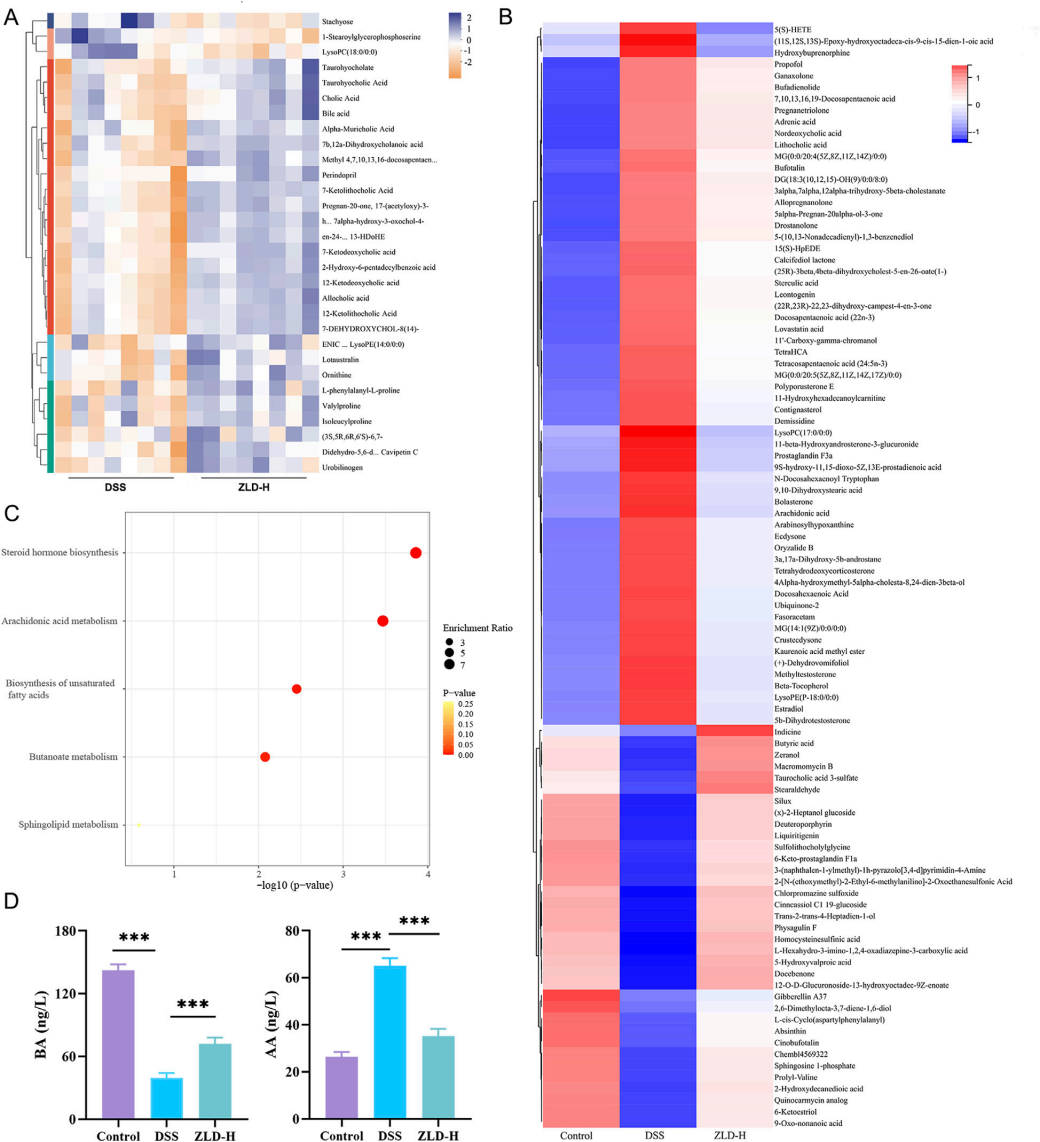
Fecal metabolite composition (n = 8 per group). **(A)** Heatmap displaying the top 30 fecal metabolites differently expressed between the DSS and ZLD-H groups in the positive ion mode. Different colors indicate different metabolite expressions; **(B)** Heatmap analysis depicting the relative levels of 96 potential biomarkers through cluster analysis; **(C)** Metabolic pathway analysis; **(D)** Expression of butyrate acid and arachidonic acid.

The top 30 metabolites, identified in both positive and negative ion modes, that exhibited differentially expressed between the DSS and ZLD-H groups, as well as between the control and DSS groups are shown in Figure 7A, Supplementary Figures S10–S12. Metabolites with VIP (Variable importance for the projection) scores> 1 and *P-*values < 0.05 were selected. This metabolite screen yielded 96 metabolites, as illustrated in Supplementary Table S6 and Figure 7B. The DSS and ZLD-H groups exhibited differential metabolites totaling 58. ZLD-H significantly enhanced the levels of 29 metabolites, including butyrate acid (BA), zeranol, macromomycin B, and taurocholic acid 3-sulfate, while significantly reducing the levels of other 29 metabolites including 5(S)-HETE, arachidonic acid (AA), tetrahydrodeoxycorticosterone, docosahexaenoic acid, estradiol, and 5b-dihydrotestosterone.

Metabolic pathway enrichment analysis using MetaboAnalyst 6.0 indicated significant alterations in five major pathways (Figure 7C). Four pathways, including steroid hormone biosynthesis, AA metabolism, unsaturated fatty acid biosynthesis, and BA metabolism, were the main enriched in these differential metabolites. Thus, ZLD treatment significantly reduced the abundance of AA, 5(S)-HETE, while increasing that of BA. Furthermore, AA metabolism was shown to be hyperactivated in UC (Wu et al., 2022), whereas the butanoate metabolism pathway was shown to be inhibited (Jia et al., 2023).

Quantitative analysis by ELISA further revealed that ZLD significantly increased the levels of BA while reducing the levels of AA (Figure 7D).

### 3.5 Association between intestinal flora and fecal metabolites

To delve deeper into the interplay between specific microbes and differential metabolites, we performed correlation analyses to reveal the coordinated changes between ZLD treatment metabolites and 7 specific microbiomes.

**FIGURE 8:**
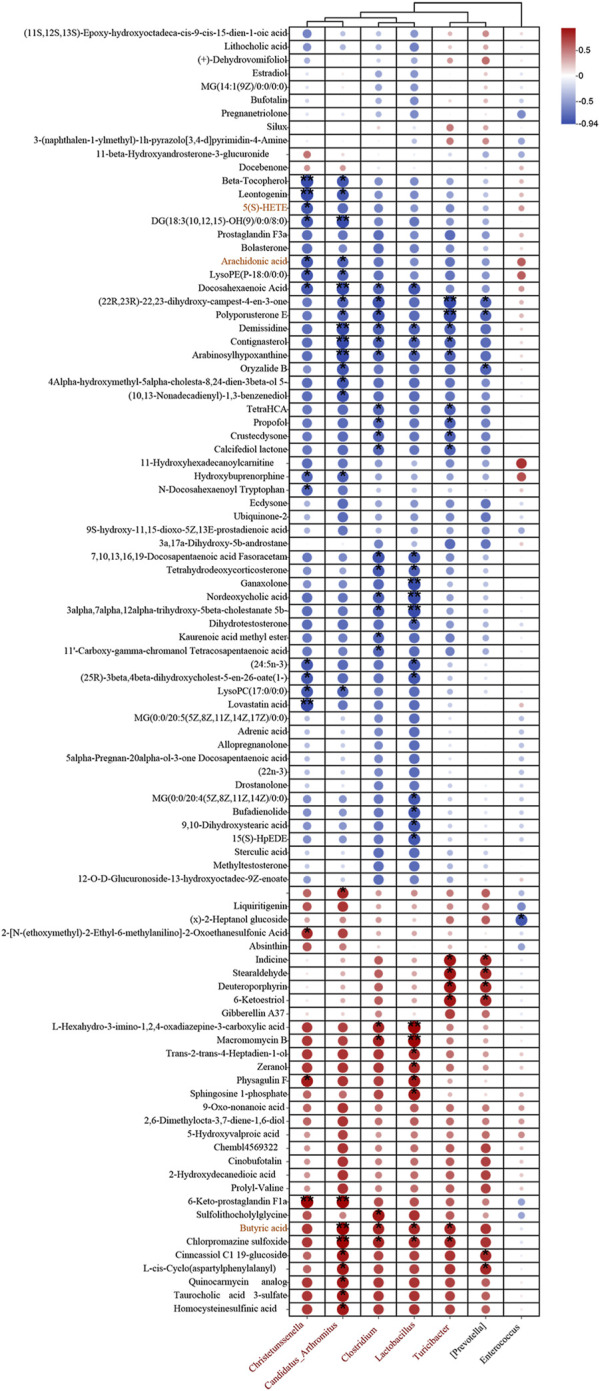
Association between markedly distinct gut microbiome (at genus) and significantly modified fecal metabolites. Fecal metabolites associated with major metabolic pathways are highlighted in red. (*p < 0.05, **p < 0.01, ***p < 0.001).

Based on the criteria of |r|>0.57 and *P*< 0.05, the correlation identified 111 pairs of significantly correlated differential flora-metabolites (*P*< 0.05), with 19 pairs of correlations being more significant (*P*< 0.01). *Christensenella*, *Clostridium*, *Candidatus_Arthromitus*, *Lactobacillus* abundance was negatively correlated with most of the elevated differential metabolites and positively correlated with most of the reduced differential metabolites in the DSS group. The ZLD-induced specific enterobacterial genera *Christensenella*, *Candidatus_Arthromitus* with AA, and *Christensenella* were negatively correlated with 5(S)-HETE; whereas *Clostridium*, *Candidatus_Arthromitus*, *Lactobacillus*, and *Turicibacter* were all positively correlated with BA (Figure 8). In accordance with these results, we hypothesized that the modulation of key metabolite levels by specific gut flora influences corresponding metabolic pathways, thereby potentially explaining the therapeutic mechanism of ZLD for UC. This involves downregulation of the levels of AA, 5(S)-HETE to inhibit the over-activity of the AA metabolic pathway in its participation, and upregulation by BA to promote the BA metabolic pathway.

### 3.6 Effect of ZLD on levels of inflammatory factors and the TLR4/NF-κB/NLRP3 signaling pathway

**FIGURE 9:**
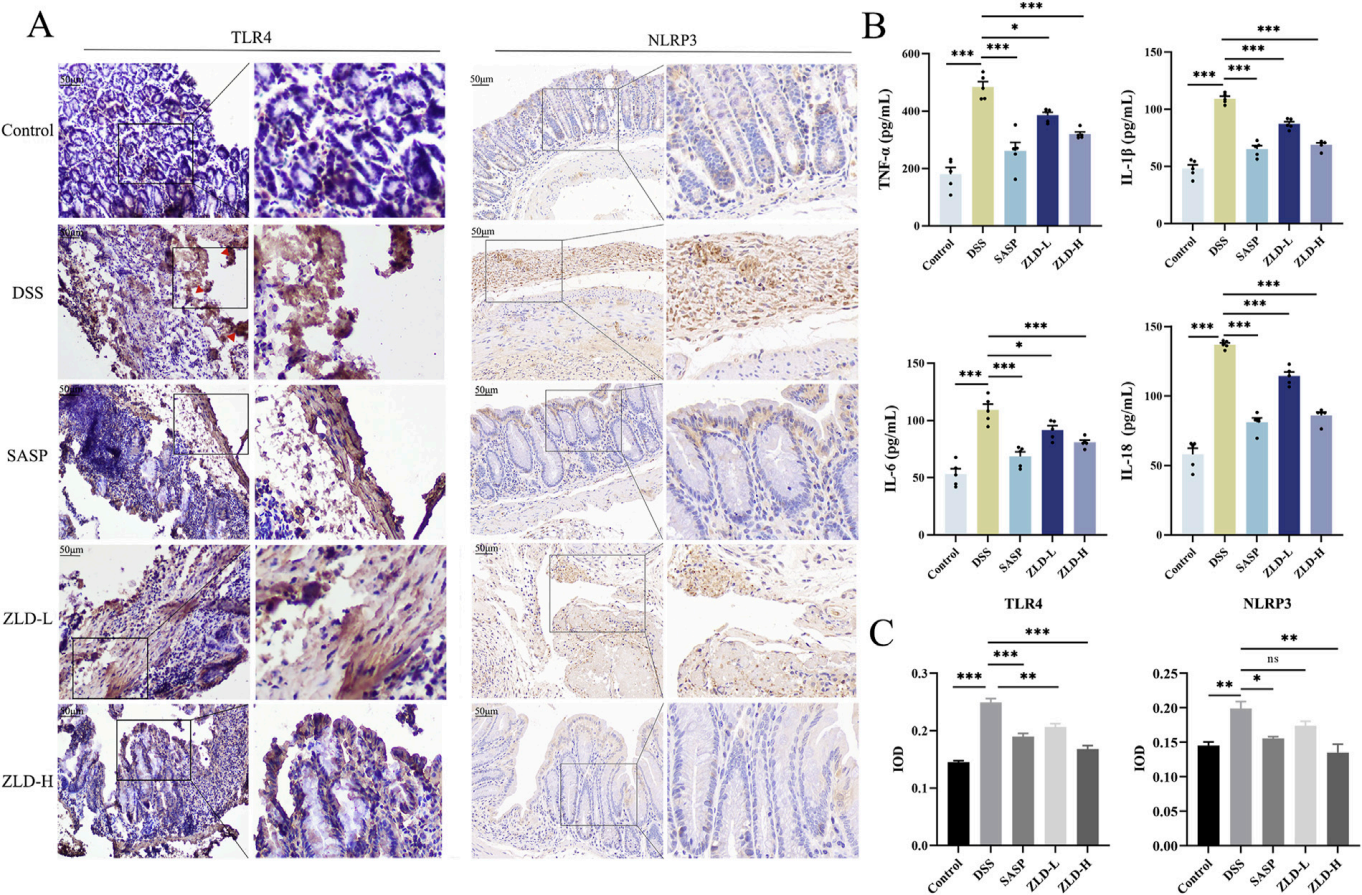
Expression of inflammatory factors, TLR4, NLRP3 in colonic tissues. **(A, C)** TLR4 and NLRP3 protein levels for colon tissues (scale bars = 500 μm); **(B)** TNF-α, IL-1β, IL-6, IL-8 levels of colon tissues; *p < 0.05, **p < 0.01, ***p < 0.001, ^ns^ *p* > 0.05.

**FIGURE 10:**
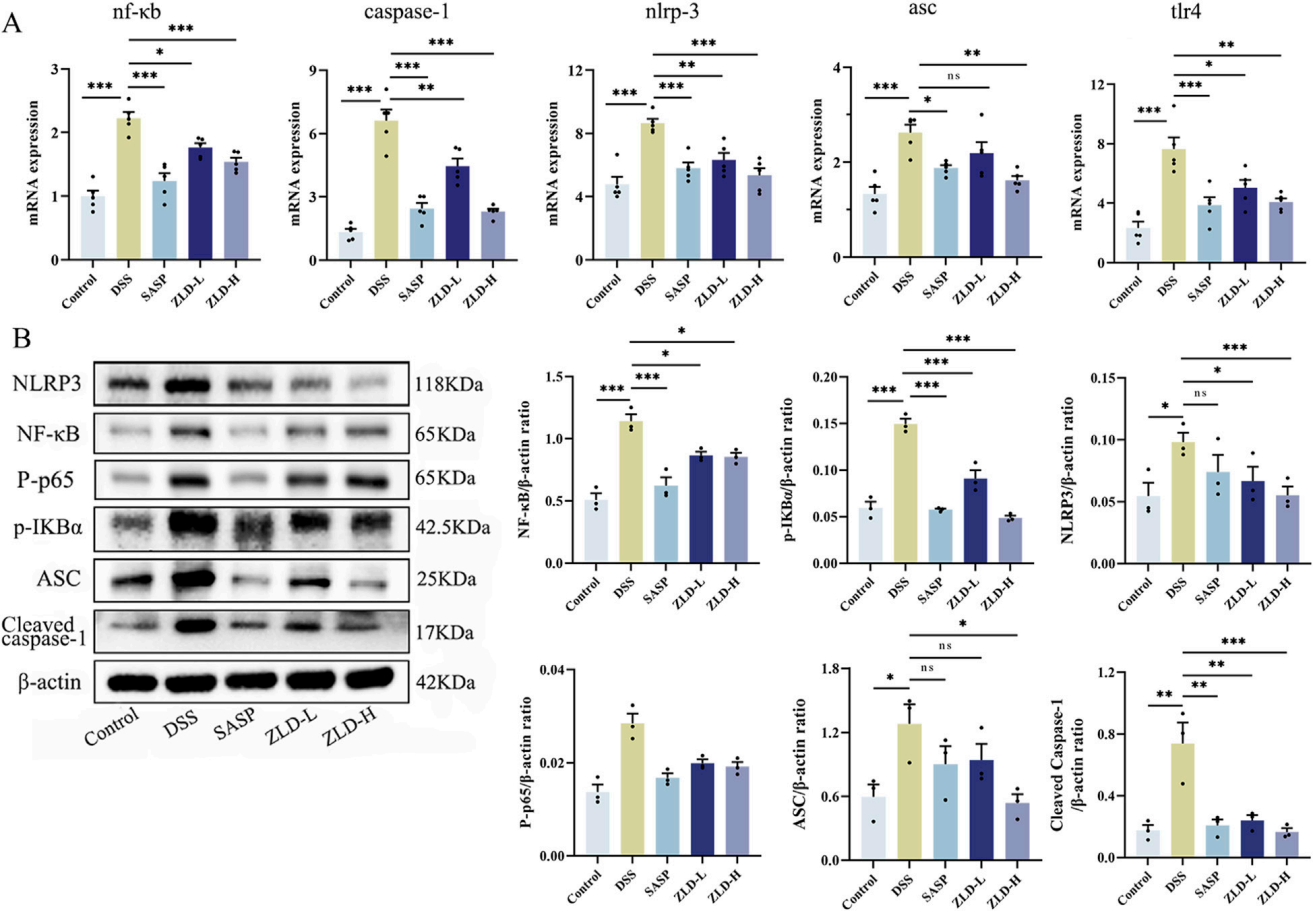
ZLD modulates inflammatory pathway **(A)** Effects on mRNA levels of nf-κb, caspase-1, nlpr3, asc and tlr4; **(B)** Effects on protein levels of p-IKBα, NF-κB p65, P-p65, cleaved caspase-1, NLRP3, and ASC; *p < 0.05, **p < 0.01, ***p < 0.001, ^ns^ *p* > 0.05).

In Figure 9B, levels of IL-6, IL-1β, IL-18, and TNF-α were markedly increased in the DSS group compared to the control group. However, the levels of these pro-inflammatory factors were reduced in the SASP, ZLD-L, and ZLD-H groups than the DSS group. This demonstrated a significant mitigates the inflammatory response by ZLD. Previous studies have highlighted a positive feedback loop between NF-κB and NLRP3 in gut inflammation (Zhang et al., 2022). IHC staining showed than the control group, that the expression of TLR4 and NLRP3 was significantly increased in the UC group (Figure 9A). However, their expression was notably reduced by SASP and ZLD-H treatment. To further elucidate the involvement of the TLR4/NF-κB/NLRP3 pathway in UC, analysis of mRNA and protein expression levels of key targets in the colons of experimental animals. RT-qPCR analysis revealed significant upregulation of genes encoding tlr4, nf-κb, caspase-1, nlrp3 and asc in the DSS group, as illustrated in Figure 10A. Treatments with SASP and ZLD mitigated this upregulation. WB analysis (Figure 10B) confirmed these findings, showing significantly increased protein concentrations of Cleaved caspase-1, NF-κB p65, P-p65, p-IKBα, NLRP3, and ASC by DSS. These changes markedly were inhibited by the SASP, ZLD-L, and ZLD-H groups.

### 3.7 Correlative analysis of inflammatory factors with differential microbiology and metabolites

**FIGURE 11:**
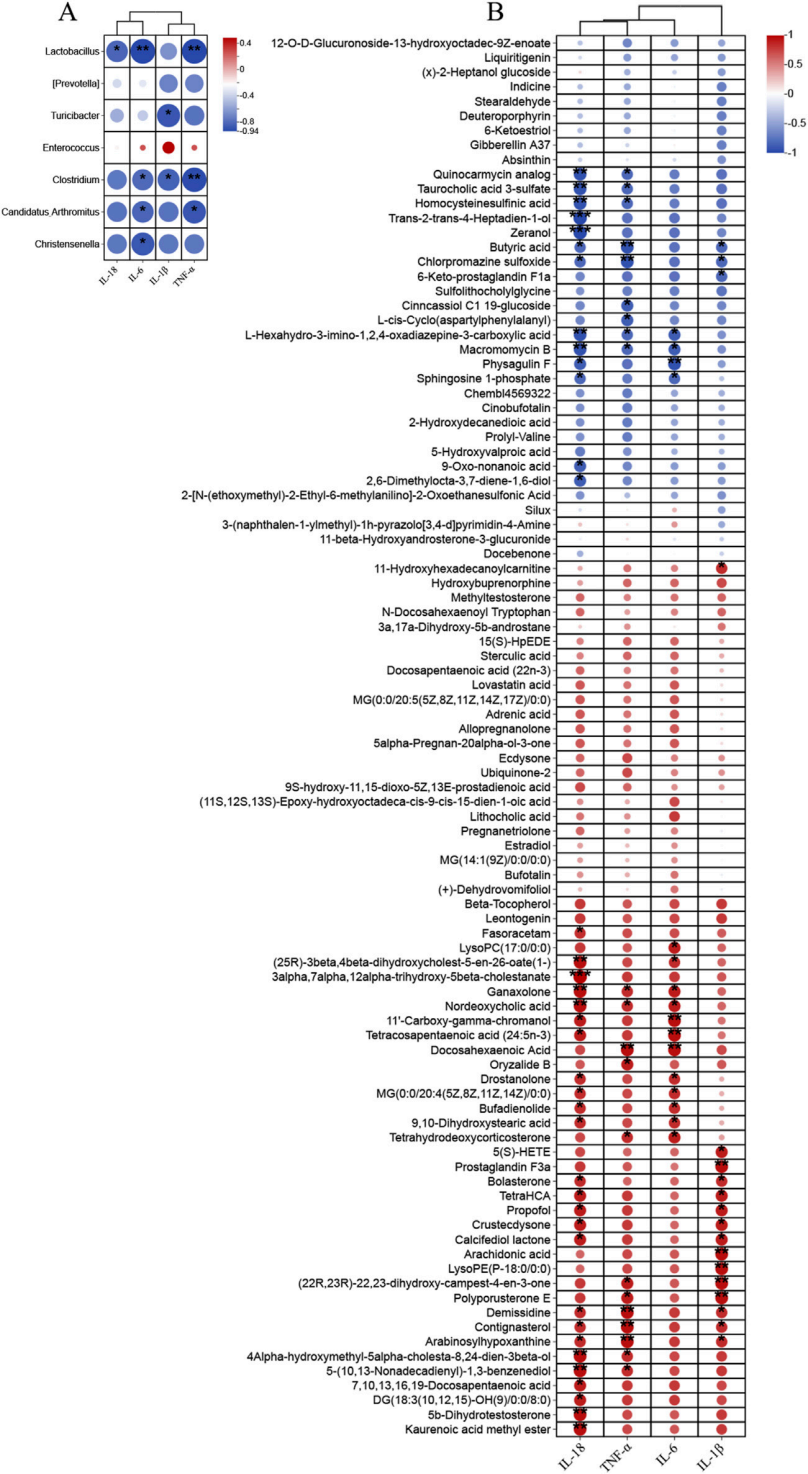
Correlation analysis was performed to examine **(A)** the relationship between differential gut microbiota (at the genus) and inflammatory factors, **(B)** the association between significantly altered fecal metabolites and inflammatory factors.

As Figure 11A showed, TNF-α was significantly negatively correlated with *Clostridium*, *Lactobacillus*, *Candidatus*_*Arthromitus*, IL-6 was significantly negatively correlated with *Clostridium*, *Christensenella*, *Lactobacillus*, *Candidatus*_*Arthromitus*. As well as IL-18 was significantly negatively correlated with *Lactobacillus* and IL-1β with *Clostridium*, *Turicibacter*. Changes in the levels of inflammatory factors were closely related to metabolites in addition to gut microbes as well. TNF-α, IL-1β, and IL-18 exhibited a negative correlation with BA, and IL-1β was positively correlated with 5(S)-HETE and AA. And TNF-α, IL-1β, IL-6 and IL-18 were associated with 22, 16, 16 and 37 metabolites, respectively (Figure 11B).

## 4 Discussion

This study highlights the therapeutic potential of ZLD in the treatment of UC, focusing on its ability to modulate gut microbiota, microbial metabolites, and inflammatory pathways. Mice treated with ZLD showed a significant improvement in disease activity with increases in body weight, reductions in colon shortening, and lower DAI scores. Histopathological improvements, including reduced colonic inflammation and enhanced intestinal barrier integrity, were evident following ZLD treatment, as observed through histological and transmission electron microscopy (TEM) analyses. Collectively, these findings indicate that ZLD effectively ameliorates disease symptoms, prevents colonic damage, and promotes intestinal barrier repair in the DSS-induced UC mouse model.

Dysbiosis of the gut microbiota has emerged as a key contributor to UC pathogenesis (Haneishi et al., 2023; Nishida et al., 2018). Mice with UC were shown to exhibit a lower abundance and number of gut microbial species than their healthy counterparts, while high microbial diversity benefits health (Jialing et al., 2020; Peng et al., 2020). Moreover, the F/B ratio is commonly used to assess gut microbiota imbalance, with studies showing alterations in this ratio in UC mice (Kabeerdoss et al., 2015; Liu Y-J et al., 2020b). One of the key findings of the present study is the modulatory effect of ZLD on gut microbiota composition. ZLD reversed the dysregulation of the F/B ratio in UC mice, reduced the abundance of pathogenic bacteria (e.g., *Enterococcus*, *Shigella, Escherichia*), and promoted the growth of beneficial bacteria, including *Lactobacillus*, *Christensenella*, and butyrate-producing *Clostridium* species. Notably, *Lactobacillus* has been shown to mitigate intestinal injury by inhibiting the release of inflammatory mediators including IL-6, IL-1β, and TNF-α, thereby alleviating intestinal inflammation (Li et al., 2023). Similarly, *Christensenella* has been reported to reduce the expression of TLR4 and NF-κB, as well as inflammatory cytokines such as IL-1β, IL-6, and TNF-α in both the liver and colon, further modulating the inflammatory response (Pan et al., 2022). These results support the well-established link between gut microbiota dysbiosis and various intestinal diseases, including UC (Cheng et al., 2023; Li et al., 2021). Collectively, these findings suggest that ZLD may exert therapeutic effects in UC by regulating gut microbiota, inhibiting the growth of pathogenic bacteria, promoting beneficial bacterial proliferation, improving intestinal microecology, and reducing the inflammatory response.

Dysbiosis of the intestinal flora not only affects the gut environment but is also closely associated with metabolic alterations. In our study, UC mice exhibited significant metabolic disruptions, and treatment with ZLD modulated gut flora metabolites, particularly by promoting butyrate acid (BA) production and inhibiting the accumulation of arachidonic acid (AA) and its metabolite 5(S)-HETE. BA, a key short-chain fatty acid, BA, a key short-chain fatty acid, has potent anti-inflammatory properties, primarily by inhibiting NF-κB activation and reducing interferon-γ production, thus mitigating inflammation (Salvi and Cowles, 2021). Additionally, BA enhances mucin production, strengthens tight junctions, and promotes epithelial cell repair and intestinal barrier restoration (Hamer et al., 2008). AA is a key precursor of inflammatory mediators and can be metabolized into leukotrienes and prostaglandins, both of which play critical roles in inflammation and immune responses. In UC, the AA pathway is activated, converting AA into the inflammatory mediator leukotriene A4 (LTA4) (Gür et al., 2018; Kim et al., 2021; Tjonneland et al., 2009). This activation triggers signaling pathways like NF-κB, promoting the expression and release of inflammatory factors, which establish a positive feedback loop that exacerbates inflammation (Serhan and Savill, 2005). Consequently, inhibiting AA metabolic pathways and their inflammatory products presents a promising therapeutic strategy for UC. Our findings demonstrate that ZLD promoted BA production and inhibited the accumulation of AA and its metabolite 5(S)-HETE, further highlighting its potential in reducing inflammation and protecting the intestinal barrier.

In this study, ZLD significantly reduced inflammatory responses by lowering the levels of TNF-α, IL-1β, IL-6, and IL-8, as well as the expression of TLR4, p-IκBα, NF-κB, caspase-1, NLRP3, and ASC proteins and genes in UC mice. These findings are consistent with previous studies, which demonstrate that the activation of NF-κB and the NLRP3 inflammasome results in elevated levels of caspase-1, IL-1β, and IL-18 (Wang et al., 2018). We propose that ZLD exerts its therapeutic effect in UC by inhibiting the TLR4/NF-κB/NLRP3 signaling pathway. The efficacy of ZLD is closely linked to its ability to block the positive feedback loop of inflammation in UC by modulating this key inflammatory cascade. Previous research has shown that intestinal epithelial cells recognize TLRs, which are critical for detecting microbial signals from the gut microbiota (Burgueño and Abreu, 2020). Specifically, intestinal microbes (e.g., *Escherichia*, *Bacteroides*, and *Enterococcus*) release LPS, which activates the TLR4/NF-κB/NLRP3 inflammasome pathway, leading to the release of pro-inflammatory cytokines, including IL-1β, TNF-α, and IL-6, thereby contributing to the pathogenesis of UC(Kawai and Akira, 2011; Afonina et al., 2017; Liu et al., 2023). Moreover, the correlations between gut microbiota composition, microbial metabolites, and inflammatory cytokines suggest that ZLD’s therapeutic effects may be mediated via the gut microbiota.

Research has highlighted the association between dysbiosis of gut microbiota, host metabolic functions, and diverse intestinal disorders such as UC(Cheng et al., 2023; Li et al., 2021). In the study, a tight association between gut microbiota and fecal metabolites was found by correlation analysis, which suggests that an imbalance of gut microbes may influence metabolic pathways. *Christensenella* was found to be associated with reduced levels of the metabolites AA, 5(S)-HETE, and *Candidatus_Arthromitus*, *Clostridium*, *Lactobacillus*, and *Turicibacter* were associated with increased levels of the metabolite BA. In this study, ZLD reduced AA and 5(S)-HETE levels and increased BA levels. AA and 5(S)-HETE were positively correlated with inflammatory factors and BA showed a correlation with inflammatory factors.

This study explored the therapeutic mechanisms of ZLD in treating UC. n conclusion, ZLD regulated UC through modulation of gut bacteria, gut bacteria-metabolite interactions, and the TLR4/NF-κB/NLRP3 signaling pathway. Correlation analysis further indicated that several significantly altered gut microbes were closely associated with changes in fecal metabolites and inflammatory factors. These findings suggest that ZLD exerts its therapeutic effects in UC via multiple pathways, including gut microbiota, metabolites, and inflammatory pathways.

## Data Availability

The original contributions presented in the study are included in the article/Supplementary Material, further inquiries can be directed to the corresponding authors.
