# Supplementary material for: Zhili decoction ameliorates ulcerative colitis by modulating gut microbiota and related metabolite, and inhibiting the TLR4/NF-κB/NLRP3 pathway
Source: Front Pharmacol. 2024 Dec 23;15:1481273. doi: 10.3389/fphar.2024.1481273 (PMC11701441; doi:10.3389/fphar.2024.1481273)
Supplement: Supplementary file 1 [file DataSheet1.docx]

| **Supplementary Table1.** Chemicals and reagents table | |
| --- | --- |
| Reagents | Company |
| Dextran sulfate sodium salt | MP Bio, Cat No.: 160110 |
| Urine fecal occult blood test kit | jiancheng Bioengineering, Cat No.: C027-1-1 |
| TNF-α ELISA kit | NeoBioscience, Cat No.: EMC102a.96 |
| IL-6 ELISA kit | NeoBioscience, Cat No.: EMKC006.96 |
| IL-1β ELISA kit | NeoBioscience, Cat No.: EHC002b.96 |
| IL-18 ELISA kit | NeoBioscience, Cat No.: EMC011.96 |
| Butyric acid ELISA kit | LIANBOKEBIO, Cat No.: LBK-M04136 |
| Arachidonic Acid/AA ELISA Kit | YOBIBIO, Cat No.: U96-1592E |
| Tissue RNA Purification Kit | EZBioscience, Cat No.: EZB-RN001-plus |
| PrimeScript™ RT reagent Kit with gDNA Eraser | Takara, Cat No.: RR047A |
| TB Green® II Premix Ex Taq | Takara, Cat No.: RR420A |
| BCA protein assay kit | Solarbio,Cat NO.: PC0020 |
| β-actin antibody | ABclonal, Cat No.: AC038 |
| TLR4 antibody | Proteintech, Cat No.: 19811-1-AP |
| Phospho-IκBα antibody | Cell Signaling Technology, Cat No.: 2859 |
| Cleaved Caspase-1 antibody | Novusbio, Cat No.: NBP3-13233 |
| ASC antibody | Novusbio, Cat No.: NBP1-78977 |
| NF-κB antibody | Novusbio, Cat No.: NB100-2176 |
| NLRP3 antibody | Novusbio, Cat No.: NBP2-12466 |
| HRP-conjugated secondary antibody | Abcam, Cat No.: ab97051 |
| OMEGA Soil DNA Kit | Omega Bio-Tek,Norcross, Cat No.: M5635-02 |
| Quant-iT PicoGreen dsDNA Assay Kit | Thermo Fisher, Invitrogen, Cat No.: P7589 |
| Tks Gflex DNA Polymerase | Takara, Cat No.: R060A |
| VAHTSTM DNA Clean Beads | Vazyme, Cat No.: N411-01 |
| MiSeq Reagent Kit v3 | Illumina, Cat No.: MS-102-3001 |

| **Supplementary Table2.** DAI Scoring Scale | | | |
| --- | --- | --- | --- |
| **Stool traits** | **Bloody stool** | **Weight loss** | **DAI score** |
| Normal | no occult blood | None | 0 |
| Mild soft stool | Occult blood weakly positive (+) | 1–5% | 1 |
| Severe soft | Occult blood positive (++) | 5–10% | 2 |
| Mild diarrhea | Occult blood strongly positive (+++) | 10–15% | 3 |
| Severe diarrhea | Bloody naked eye | > 15% | 4 |

| **Supplementary Table3.** Criteria for Geboes scoring | |
| --- | --- |
| **Structural (architectural changes)** | **Continuous Geboes** |
| No abnormality | 0 |
| Mild abnormality | 1 |
| Mild or moderate diffuse or multifocal abnormalities | 2 |
| Severe diffuse or multifocal abnormalities | 3 |
| **Chronic inflammatory infiltrate** | **Grade 1** |
| No increase | 3 |
| Mild but unequivocal increase | 4 |
| Moderate increase | 5 |
| Marked increase | 6 |
| **Lamina propria neutrophils and eosinophils** | **Grade 2** |
|  | **2A Eosinophils** |
| No increase | 6 |
| Mild but unequivocal increase | 7 |
| Moderate increase | 8 |
| Marked increase | 9 |
|  | **2B Neutrophils** |
| No increase | 9 |
| Mild but unequivocal increase | 10 |
| Moderate increase | 11 |
| Marked increase | 12 |
| **Neutrophils in epithelium** | **Grade 3** |
| None | 12 |
| < 5 % Crypts involved | 13 |
| < 50 % Crypts involved | 14 |
| > 50 % Crypts involved | 15 |
| **Crypt destruction** | **Grade 4** |
| None | 15 |
| Probable - local excess of neutrophils in part of crypt | 16 |
| Probable - marked attenuation | 17 |
| Unequivocal crypt destruction | 18 |
| **Erosion or ulceration** | **Grade5** |
| No erosion, ulceration or granulation tissue | 18 |
| Recovering epithelium + adjacent inﬂammation | 19 |
| Probable erosion focally stripped | 20 |
| Unequivocal erosion | 21 |
| Ulcer or granulation tissue | 22 |

**Supplementary Table4.** Primer Sequences for PCR

| **Primer sequence** | | |
| --- | --- | --- |
| β-actin | Forward | 5'-GTGCTATGTTGCTCTAGACTTCG-3' |
|  | Reverse | 5'-ATGCCACAGGATTCCATACC-3' |
| NF-κB | Forward | 5'-ACTGCCGGGATGGCTACTAT-3' |
|  | Reverse | 5'-TCTGGATTCGGCTAATGG-3' |
| Tlr4 | Forward | 5'-TATCGGTGGTCAGTGTGCTT-3' |
|  | Reverse | 5'-CTCGTTTCTCACCCAGTCCT-3' |
| NLRP3 | Forward | 5'-CAAGGCTGCTATCTGGAGGAA-3' |
|  | Reverse | 5'-TGCAACGGACACTCGTCATC-3' |
| ASC | Forward | 5'-GCTACTATCTGGAGTCGTATGGC-3' |
|  | Reverse | 5'-GACCCTGGCAATGAGTGCTT-3' |
| Caspase-1 | Forward | 5'-AATACAACCACTCGTACACGTC-3' |
|  | Reverse | 5'-AGCTCCAACCCTCGGAGAAA-3' |


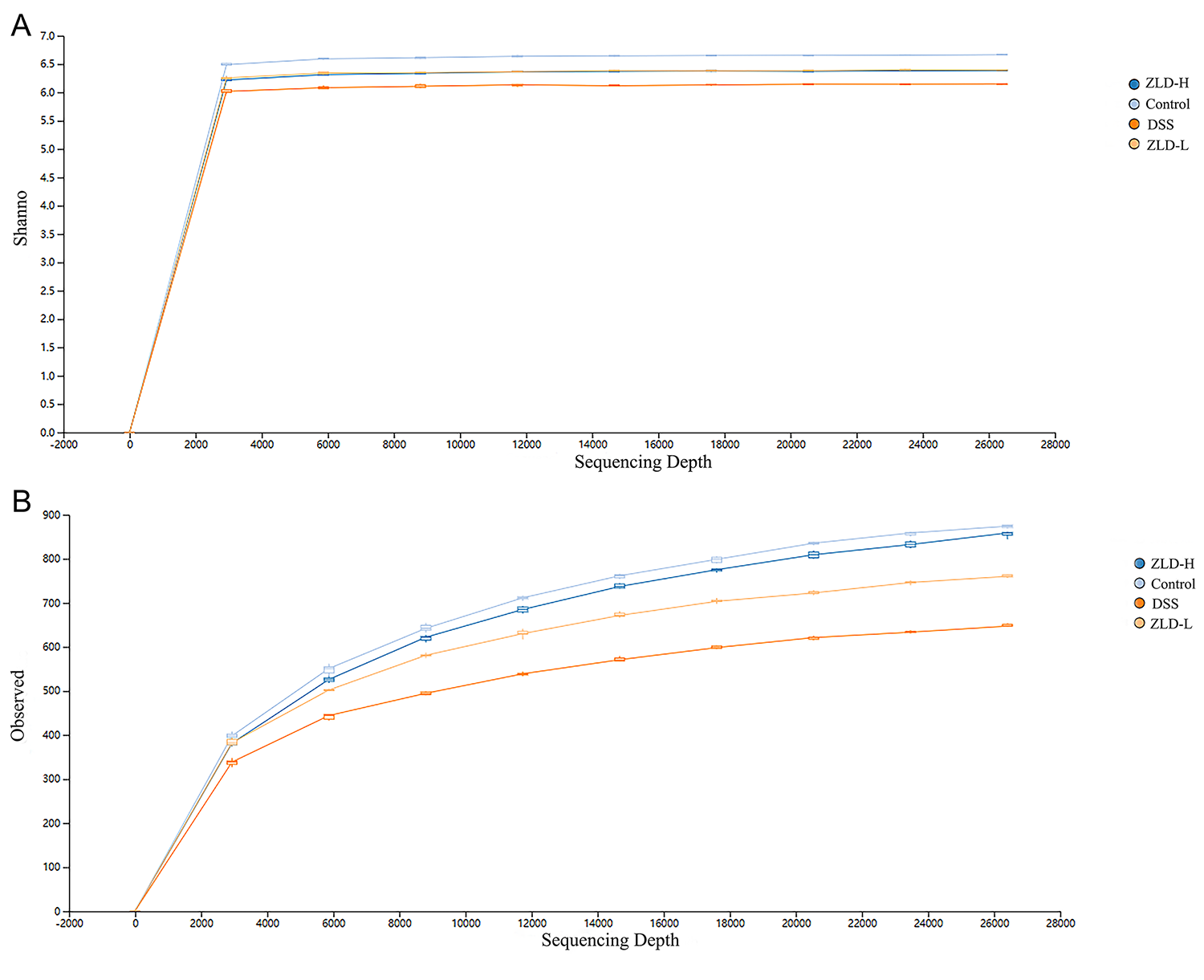


**Supplementary Figure S1.** Rarefaction curves for (A) the Shannon index and (B) Good’s coverage.


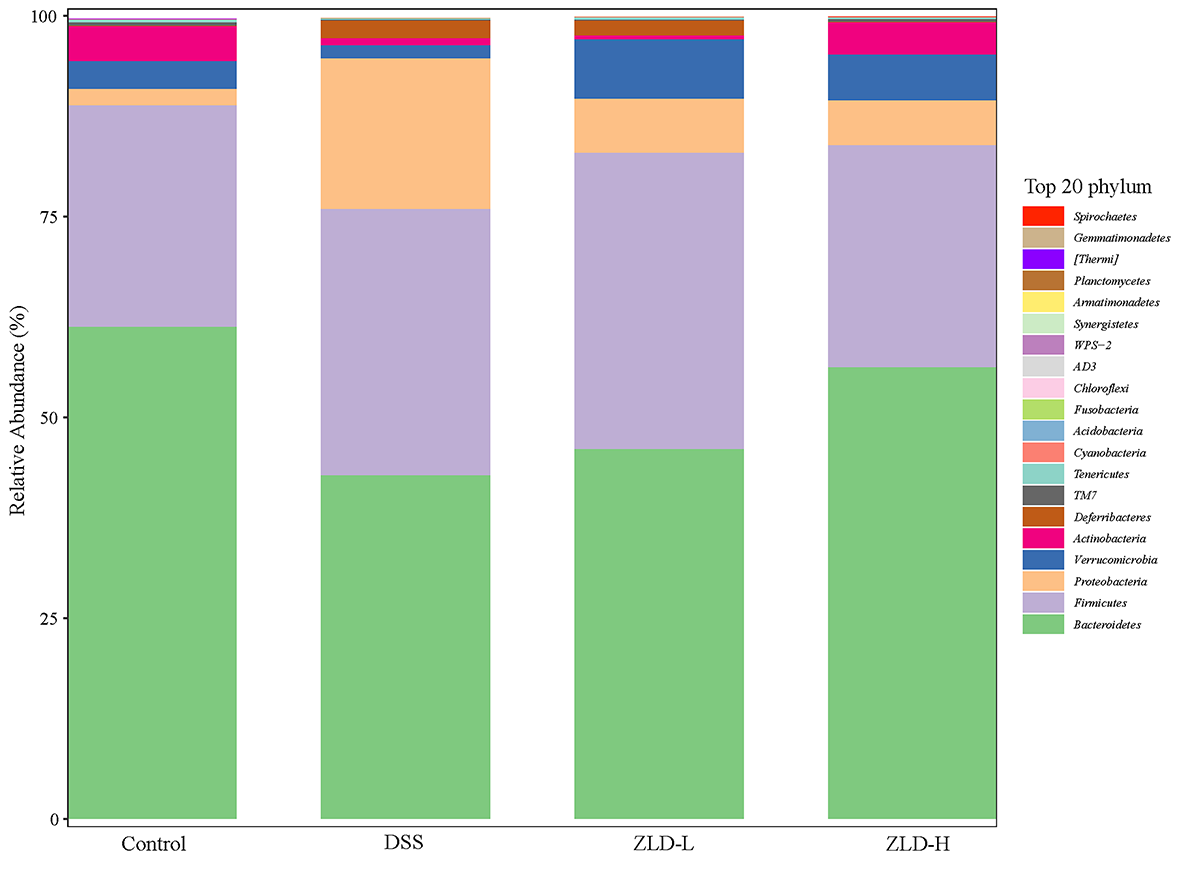


**Supplementary Figure S2.** Phylum level information on the samples.

**
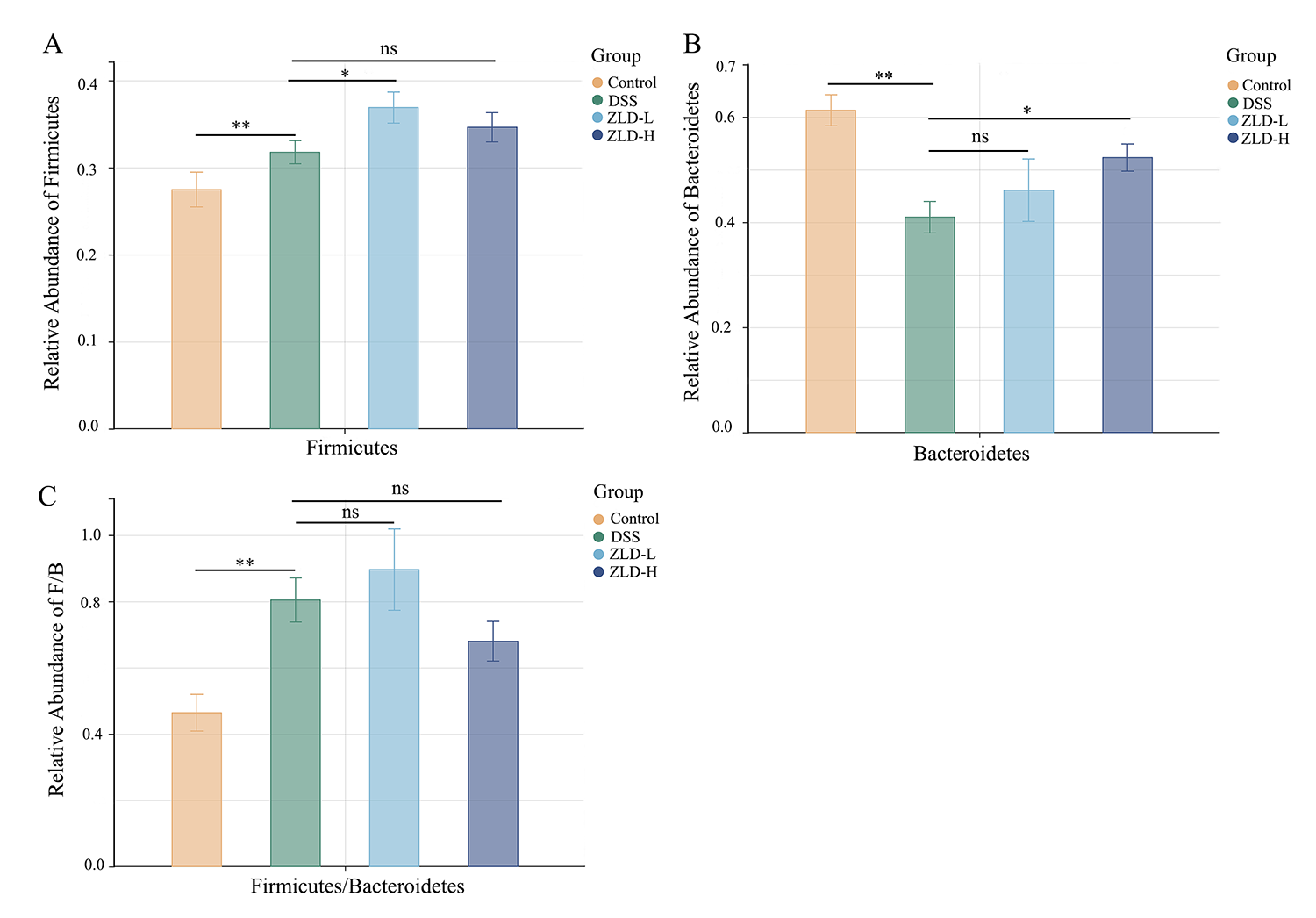
**

**Supplementary Figure S3.** Changes in Firmicutes (A), Bacteroidetes (B), Firmicutes/Bacteroidetes (C), and other bacteria. ^*^*P* < 0.05, ^*^*P* < 0.01, ^***^*P* < 0.001, ns *P* > 0.05.

**
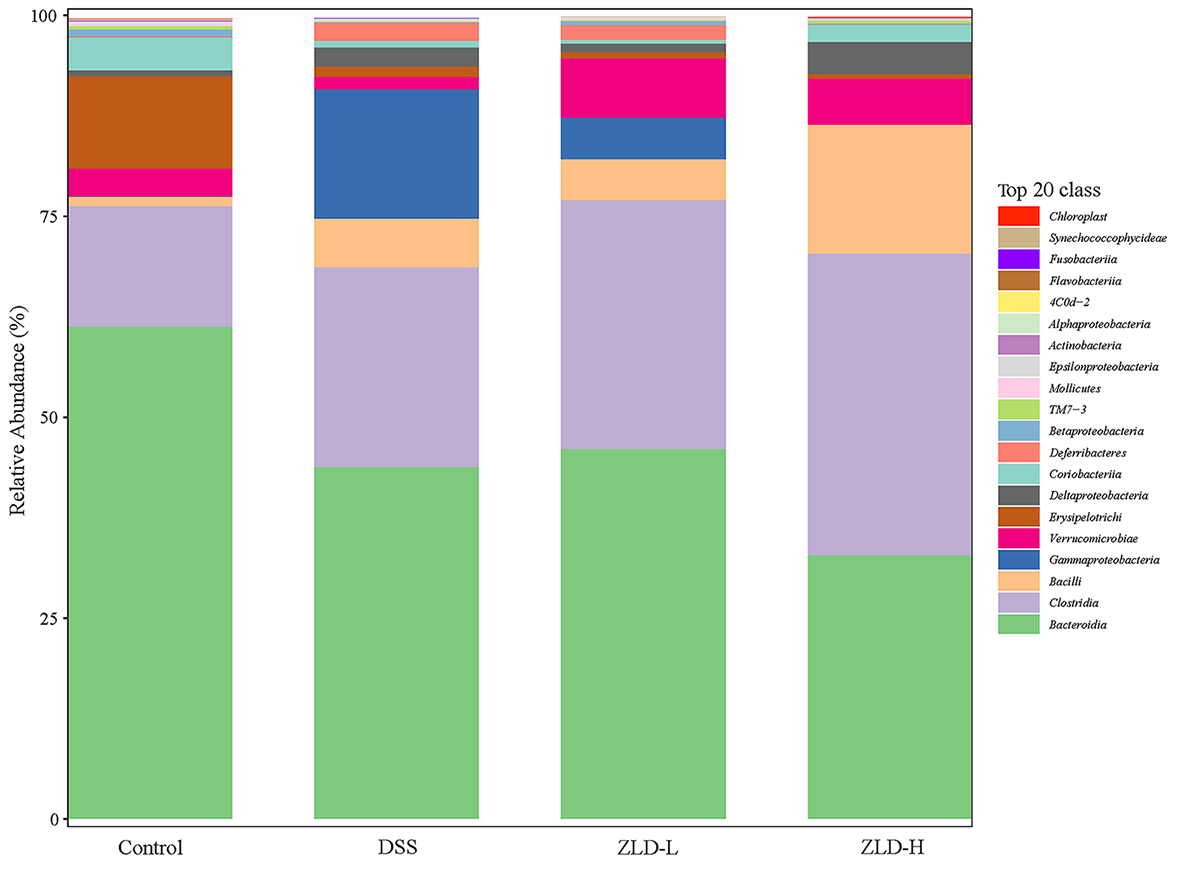
**

**Supplementary Figure S4.** Class level information on the samples.

**
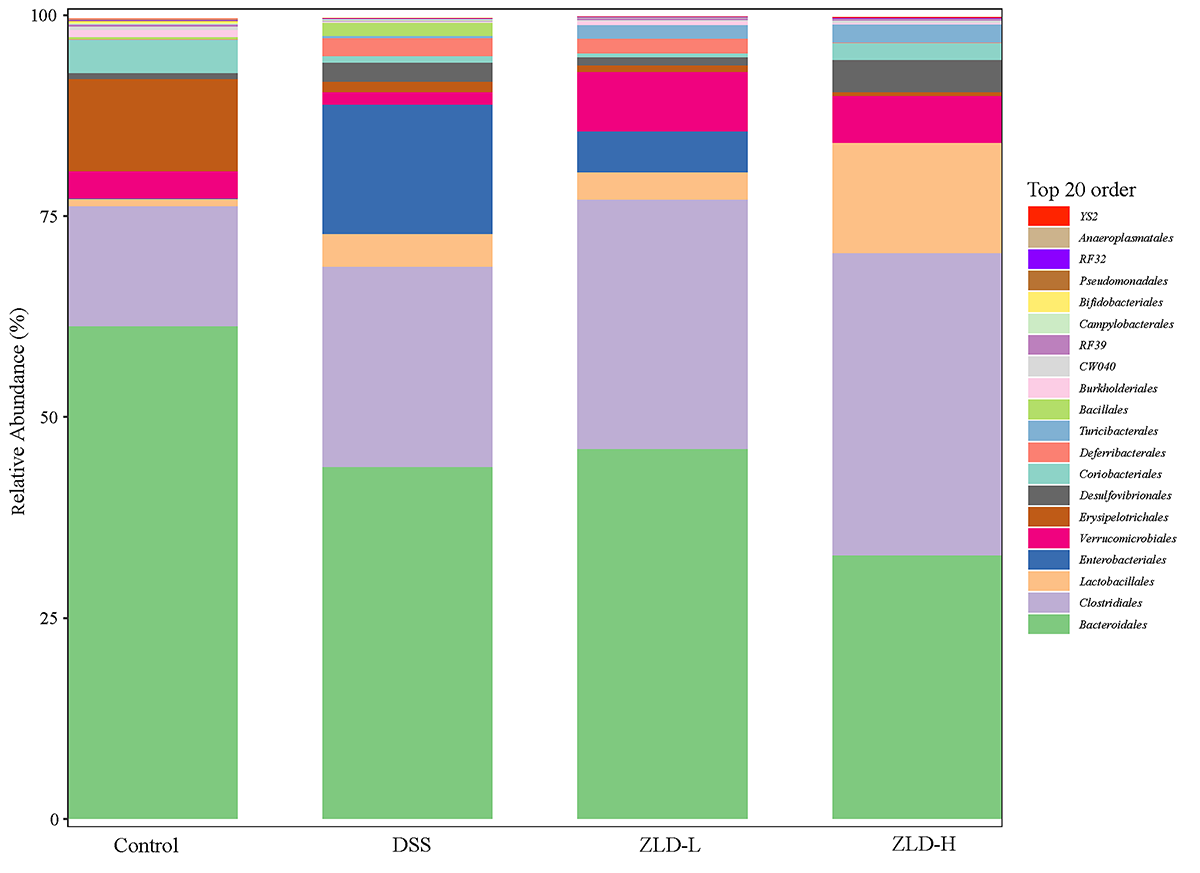
**

**Supplementary Figure S5.** Order level information on the samples.


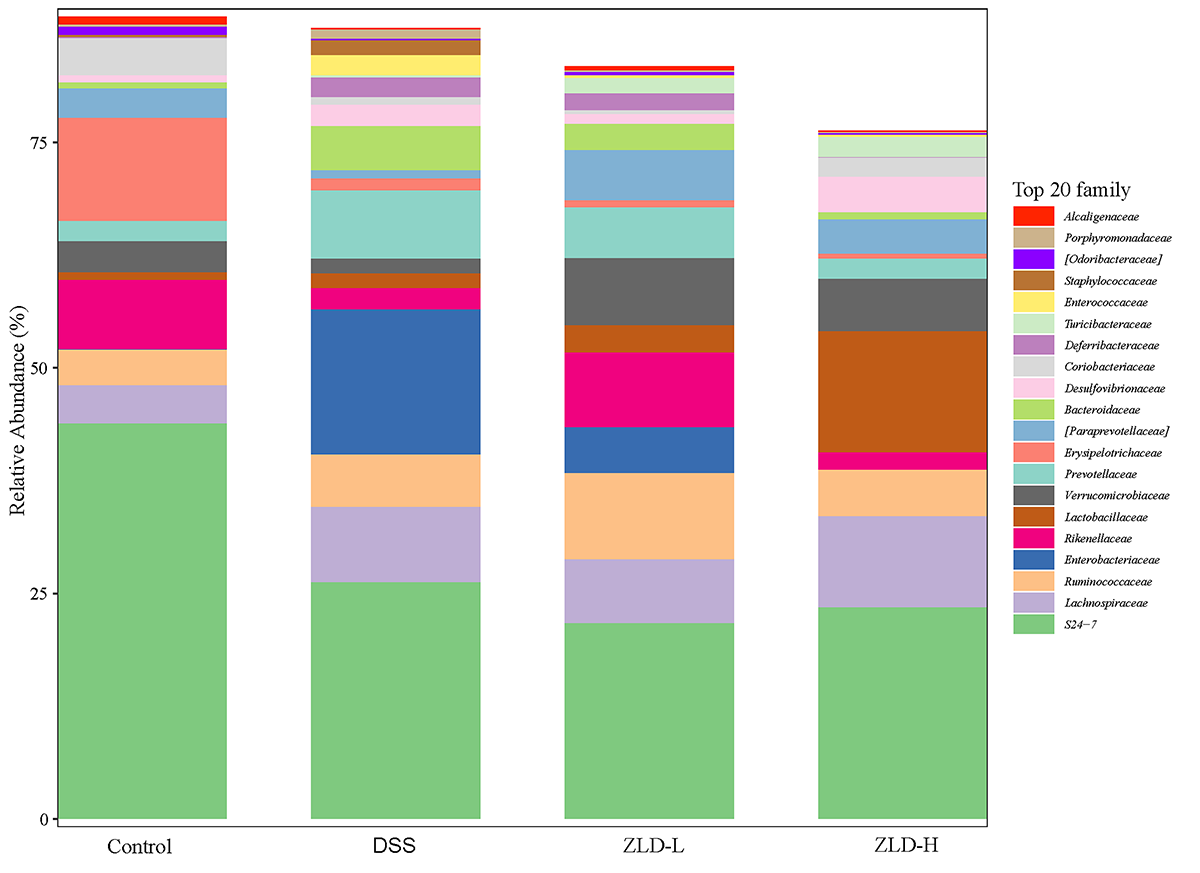


**Supplementary Figure S6.** Family level information on the samples.


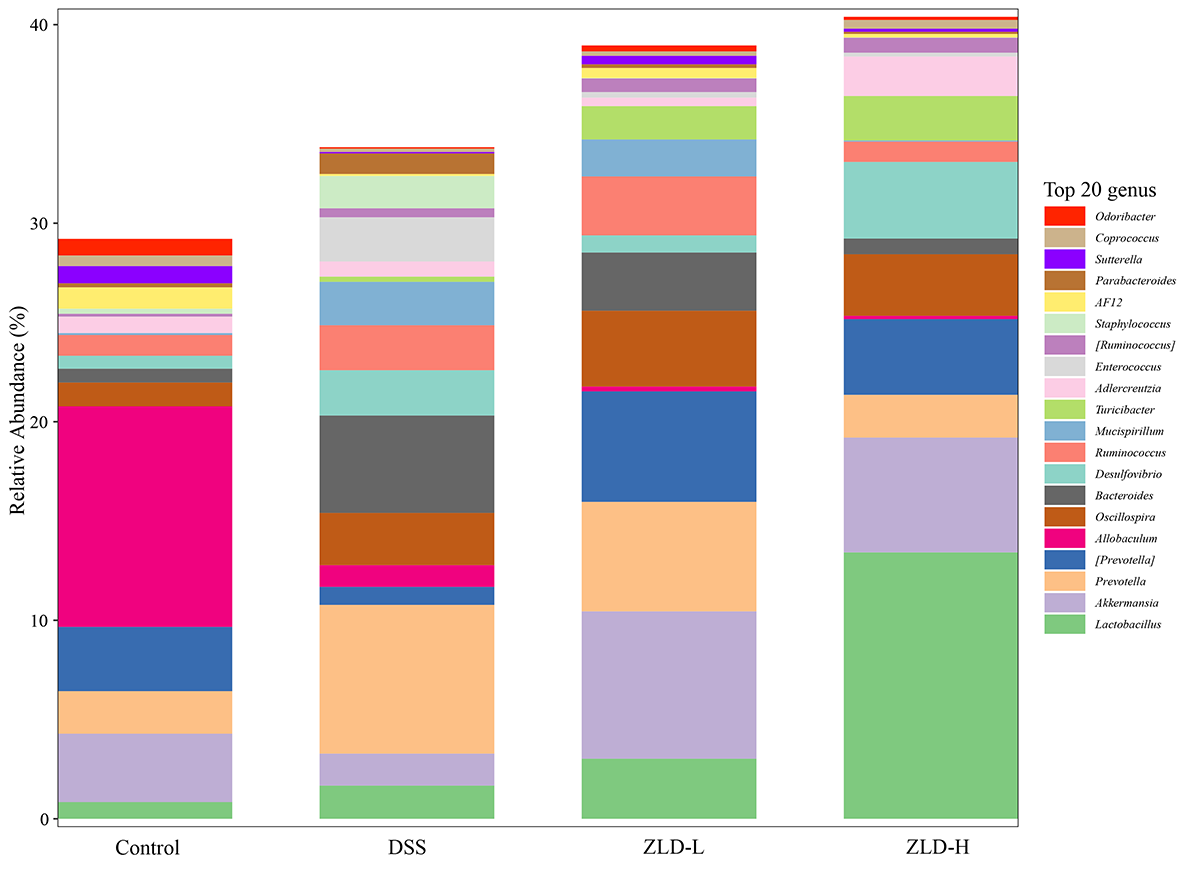


**Supplementary Figure S7.** Genus level information on the samples.


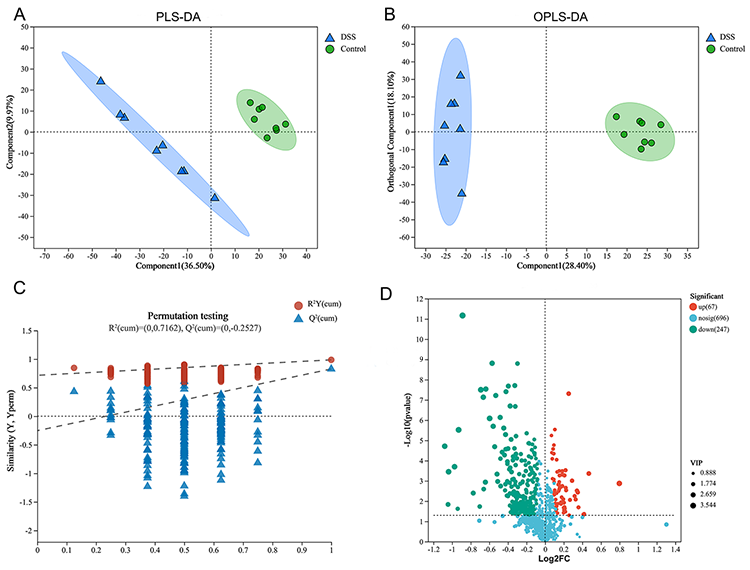


**Supplementary Figure S8.** Metabolite differences between the DSS and Control groups in a positive model. (A) PCA analysis, (B) OPLS-DA analysis, and the (C) permutation test. (D) Volcano plots of differentially expressed metabolites.


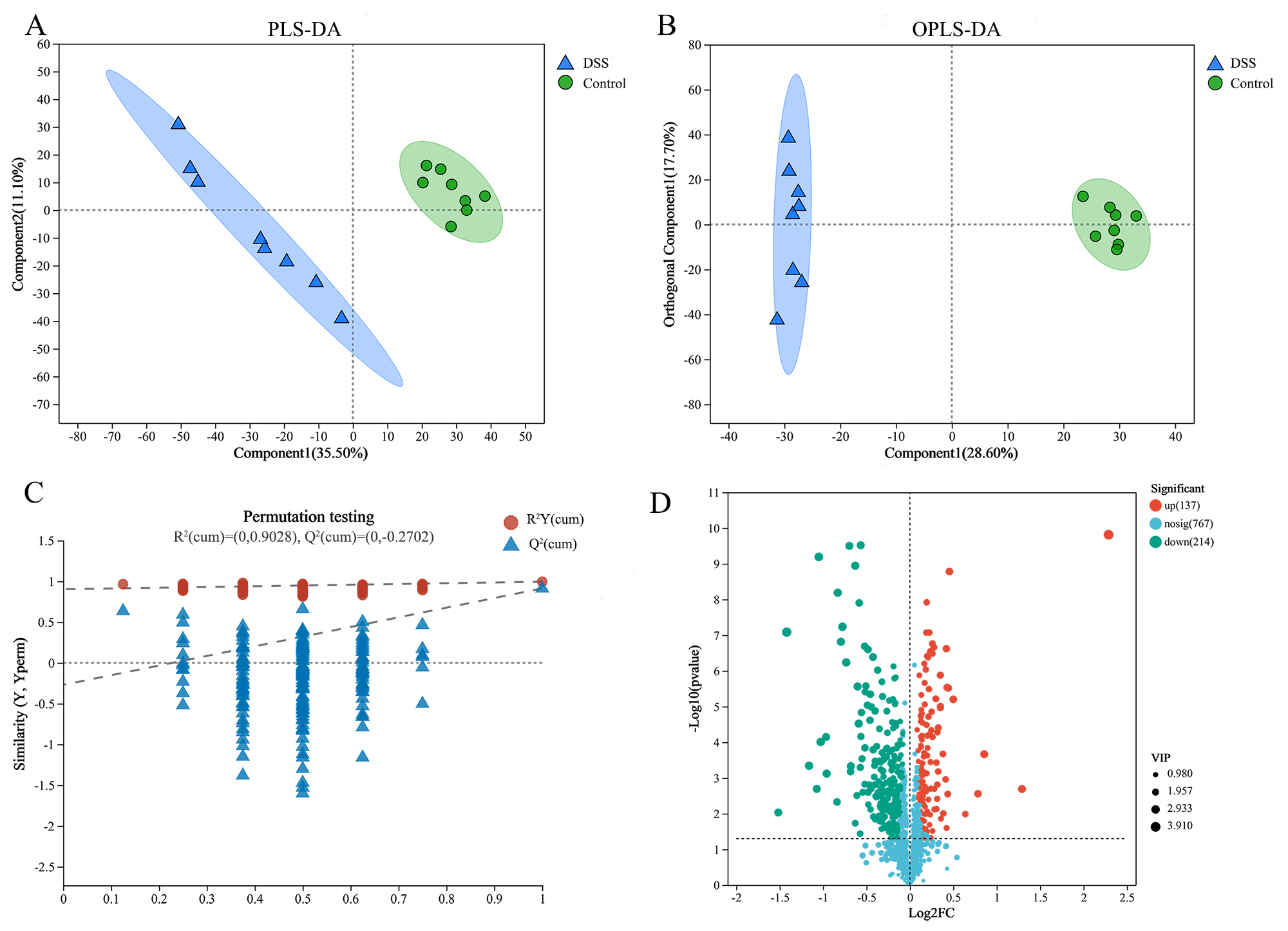


**Supplementary Figure S9.** Metabolite differences between the DSS and Control groups in a negative model. (A) PCA analysis, (B) OPLS-DA analysis, and the (C) permutation test. (D) Volcano plots of differentially expressed metabolites.


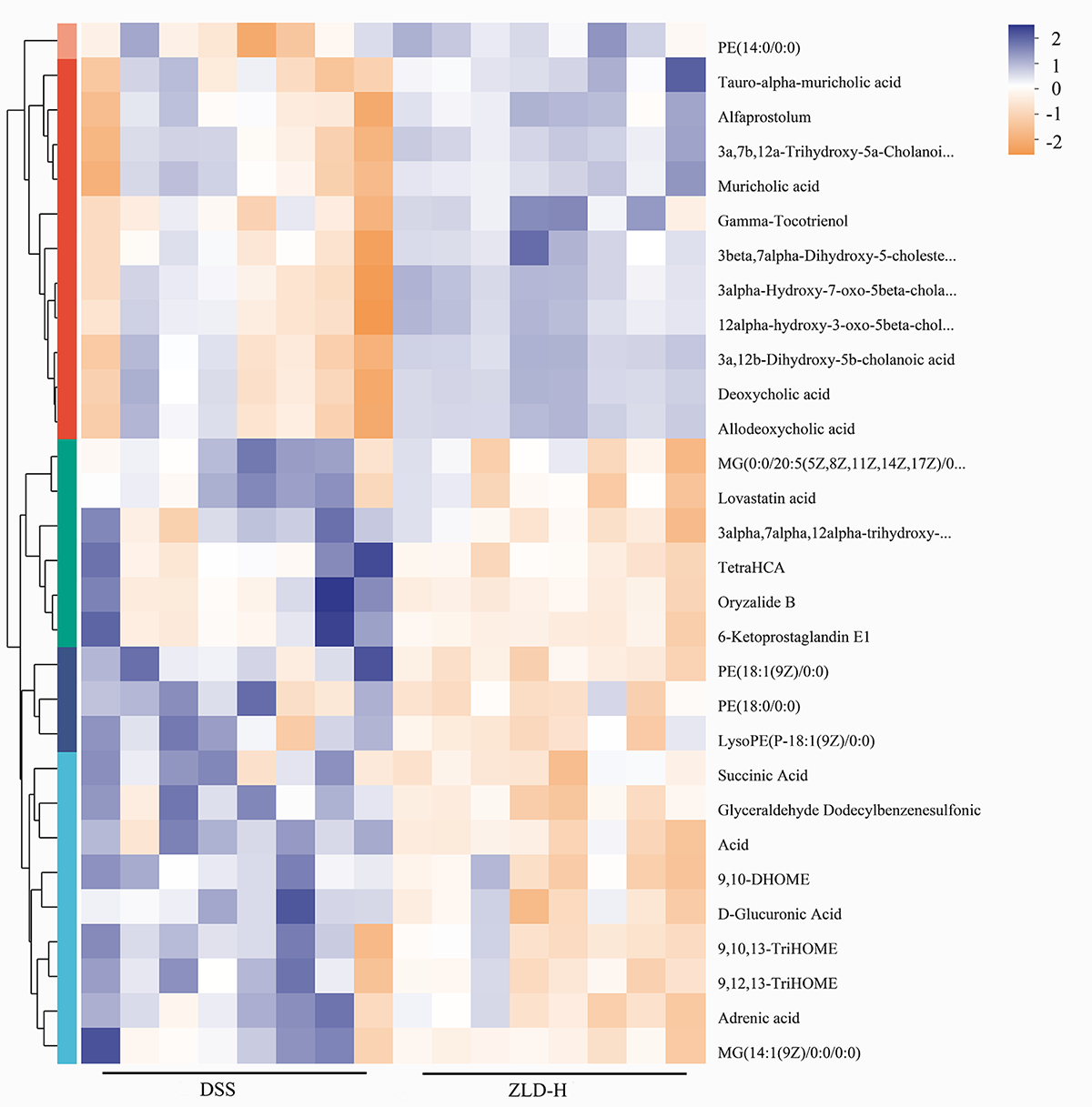


**Supplementary Figure S10.** Heatmap displaying the different fecal metabolites in the DSS and ZLD-H groups in the negative ion mode (Top30). Different colors indicate different metabolite expressions (n = 8 per group).


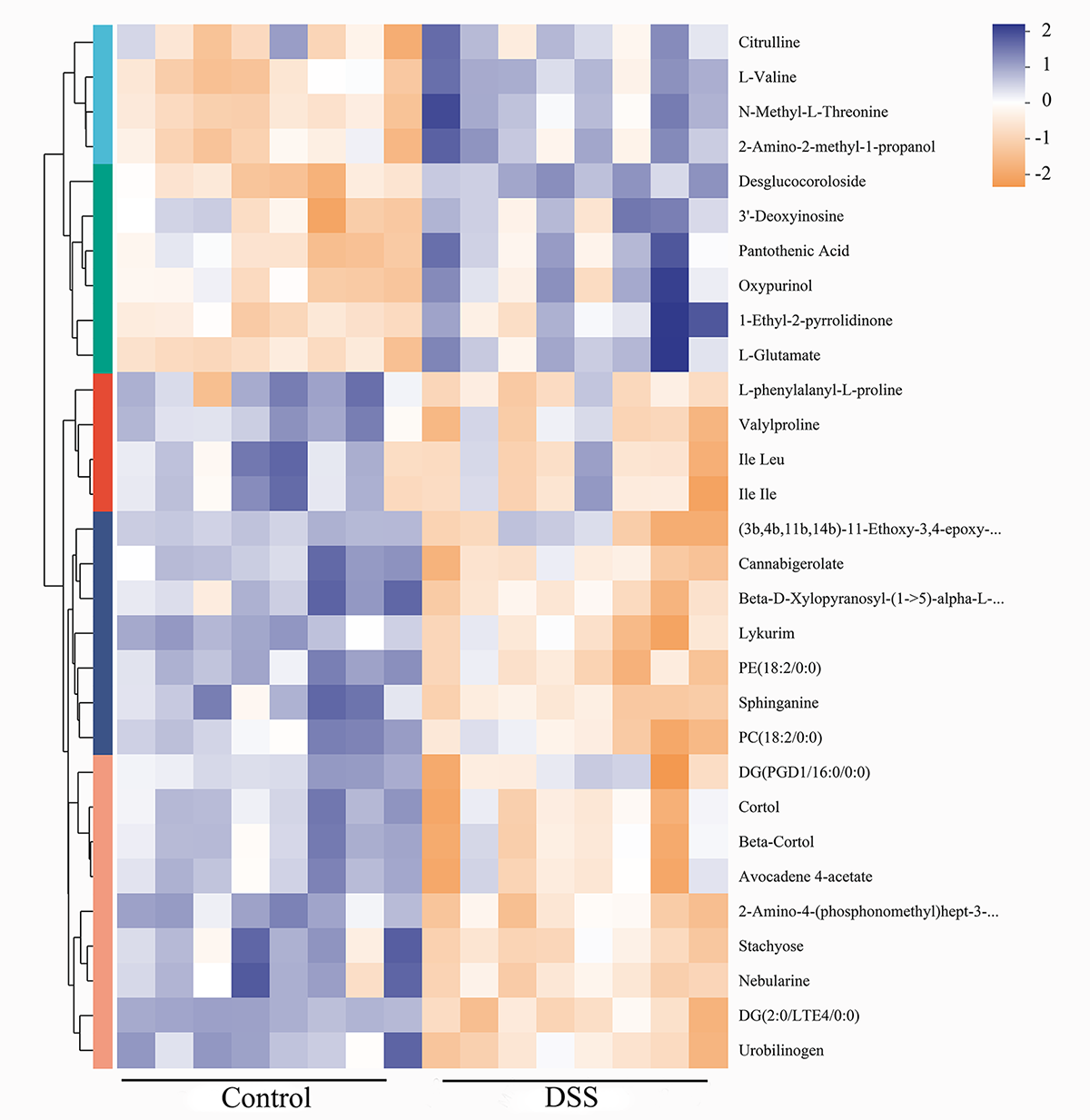


**Supplementary Figure S11.** Heatmap displaying the different fecal metabolites in the Control and DSS groups in the positive ion mode (Top30). Different colors indicate different metabolite expressions (n = 8 per group).


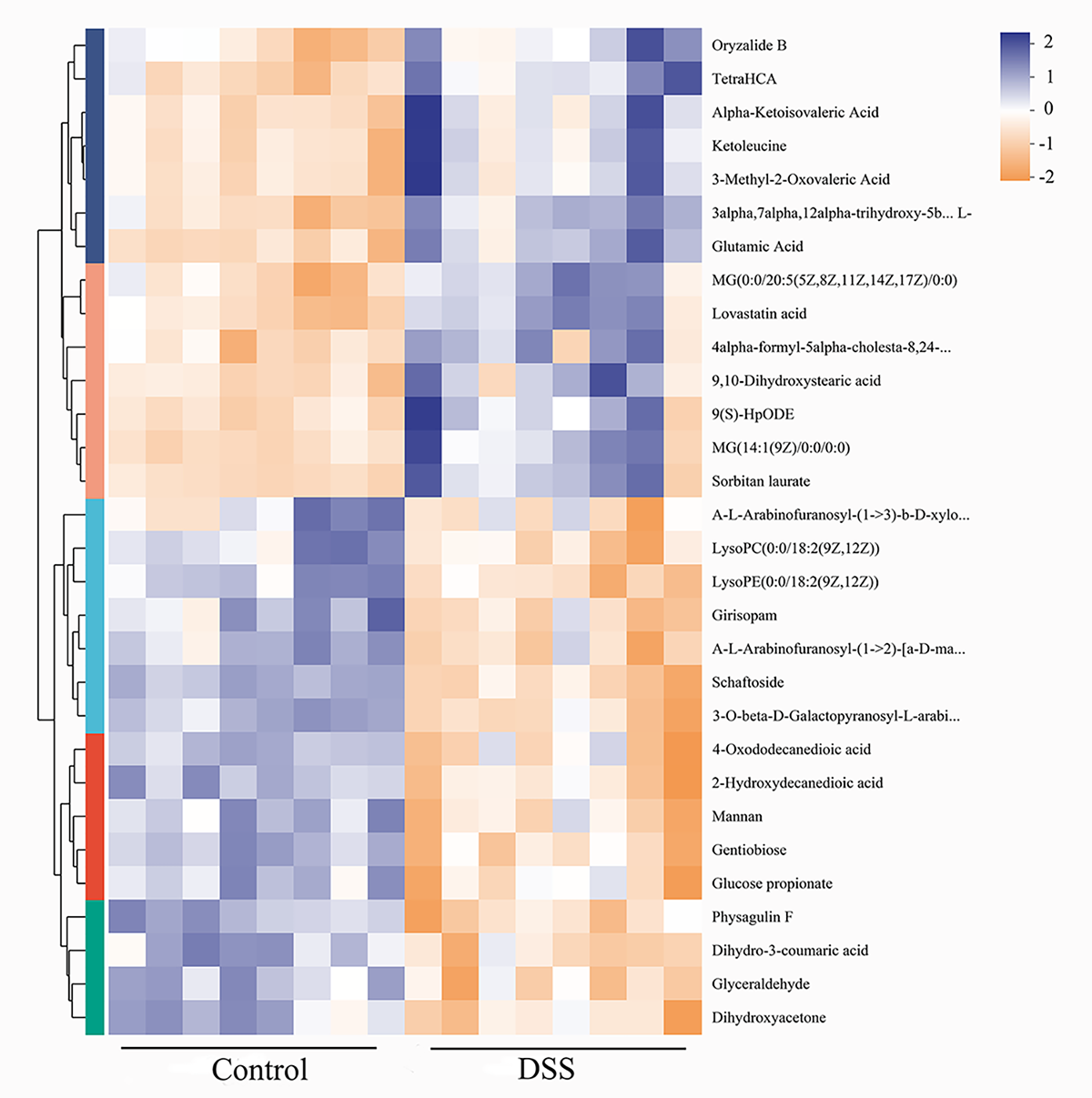


**Supplementary Figure S12.** Heatmap displaying the different fecal metabolites in the Control and DSS groups in the negative ion mode (Top30). Different colors indicate different metabolite expressions (n = 8 per group).

| **Supplementary Table5.** Chemical analysis of ZLD compound   \| **No.** \| **Compound** \| **Formula** \| **RT** \| **Reference Ion** \| **Calc. MW** \| **m/z** \| **ppm** \| **Sorce** \| \| --- \| --- \| --- \| --- \| --- \| --- \| --- \| --- \| --- \| \| 1 \| (-)-Sinoacutine \| C19 H21 N O4 \| 3.808 \| [M+H]+1 \| 327.14308 \| 328.15036 \| -12.16 \| Full match \| \| 2 \| (+)-Corynoline \| C21 H21 N O5 \| 4.898 \| [M+H]+1 \| 367.1375 \| 368.14477 \| -12.19 \| Full match \| \| 3 \| 1-Deoxynojirimycin \| C6 H13 N O4 \| 3.219 \| [M+H]+1 \| 163.08731 \| 164.09459 \| 17.51 \| Full match \| \| 4 \| 2-Hydroxy-4-methoxybenzaldehyde \| C8 H8 O3 \| 4.597 \| [M+H]+1 \| 152.04554 \| 153.05272 \| -11.88 \| Full match \| \| 5 \| 3,4-Dihydroxyphenylethanol \| C8 H10 O3 \| 4.448 \| [M-H]-1 \| 154.06138 \| 153.05411 \| -10.45 \| Full match \| \| 6 \| 3,5-Dimethoxy-4-hydroxybenzaldehyde \| C9 H10 O4 \| 4.663 \| [M+H]+1 \| 182.05579 \| 183.06306 \| -11.65 \| Full match \| \| 7 \| 3,5-Dimethoxy-4-hydroxybenzaldehyde \| C9 H10 O4 \| 5.682 \| [M-H]-1 \| 182.05643 \| 181.04915 \| -8.15 \| Full match \| \| 8 \| 4-Hydroxybenzoic acid \| C7 H6 O3 \| 5.657 \| [M-H]-1 \| 138.02998 \| 137.02271 \| -12.4 \| Full match \| \| 9 \| 4-Methyl-6,7-dihydroxycoumarin \| C10 H8 O4 \| 2.284 \| [M-H]-1 \| 192.04086 \| 191.03359 \| -7.26 \| Full match \| \| 10 \| 5-Acetylsalicylic acid \| C9 H8 O4 \| 5.802 \| [M-H]-1 \| 180.04064 \| 179.03358 \| -8.97 \| Full match \| \| 11 \| 5-Hydroxymethylfurfural \| C6 H6 O3 \| 1.237 \| [M+H]+1 \| 126.03027 \| 127.03757 \| -11.27 \| Full match \| \| 12 \| 6-Gingerol \| C17 H26 O4 \| 6.107 \| [M-H]-1 \| 294.18291 \| 293.17563 \| -0.69 \| Full match \| \| 13 \| 7-Hydroxycoumarin \| C9 H6 O3 \| 2.319 \| [M-H]-1 \| 162.03013 \| 161.02286 \| -9.64 \| Full match \| \| 14 \| 7-Methoxycoumarin \| C10 H8 O3 \| 13.216 \| [M+H]+1 \| 176.04521 \| 177.05248 \| -12.15 \| Full match \| \| 15 \| 8-O-Acetyl shanzhiside methyl ester \| C19 H28 O12 \| 3.636 \| [M+FA-H]-1 \| 448.15753 \| 493.1557 \| -1.21 \| Full match \| \| 16 \| Abscisic acid \| C15 H20 O4 \| 5.646 \| [M-H]-1 \| 264.13579 \| 263.12851 \| -1.41 \| Full match \| \| 17 \| Acetophenone \| C8 H8 O \| 1.53 \| [M+H]+1 \| 120.0563 \| 121.06358 \| -10.13 \| Full match \| \| 18 \| Adenine \| C5 H5 N5 \| 1.521 \| [M-H]-1 \| 135.05292 \| 134.04546 \| -11.66 \| Full match \| \| 19 \| Anisic aldehyde \| C8 H8 O2 \| 3.956 \| [M+H]+1 \| 136.05078 \| 137.05806 \| -12.13 \| Full match \| \| 20 \| Aristolone \| C15 H22 O \| 13.237 \| [M+H]+1 \| 218.16414 \| 219.17142 \| -13.41 \| Full match \| \| 21 \| Asperulosidic acid \| C18 H24 O12 \| 4.61 \| [M-H]-1 \| 432.12498 \| 431.11771 \| -4.15 \| Full match \| \| 22 \| Azelaic acid \| C9 H16 O4 \| 5.601 \| [M-H]-1 \| 188.10347 \| 187.09619 \| -7.4 \| Full match \| \| 23 \| Bavachin \| C20 H20 O4 \| 5.181 \| [M-H]-1 \| 324.13164 \| 323.12436 \| -13.95 \| Full match \| \| 24 \| Berberine \| C20 H17 N O4 \| 8.755 \| [M+H]+1 \| 335.11131 \| 336.11859 \| -13.26 \| Full match \| \| 25 \| Betaine \| C5 H11 N O2 \| 1.216 \| [M+H]+1 \| 117.07777 \| 118.08505 \| -10.35 \| Full match \| \| 26 \| Brevifolincarboxylic acid \| C13 H8 O8 \| 4.052 \| [M+H]+1 \| 292.01816 \| 293.02548 \| -12.87 \| Full match \| \| 27 \| Caffeic acid \| C9 H8 O4 \| 5.089 \| [M-H]-1 \| 180.04088 \| 179.0336 \| -7.64 \| Full match \| \| 28 \| Camphor \| C10 H16 O \| 4.587 \| [M+H]+1 \| 152.11831 \| 153.12561 \| -11.86 \| Full match \| \| 29 \| Chlorogenic acid \| C16 H18 O9 \| 3.996 \| [M+Na]+1 \| 354.09048 \| 377.07947 \| -13 \| Full match \| \| 30 \| Cinnamic acid \| C9 H8 O2 \| 4.001 \| [M+H]+1 \| 148.05065 \| 149.05791 \| -12.03 \| Full match \| \| 31 \| Citric acid \| C6 H8 O7 \| 4.936 \| [M-H]-1 \| 192.02449 \| 191.01839 \| -13.08 \| Full match \| \| 32 \| Coixol \| C8 H7 N O3 \| 3.202 \| [M+H]+1 \| 165.04073 \| 166.048 \| -11.3 \| Full match \| \| 33 \| Corilagin \| C27 H22 O18 \| 4.946 \| [M-H]-1 \| 634.07991 \| 633.07263 \| -1.11 \| Full match \| \| 34 \| Coumarin \| C9 H6 O2 \| 4.341 \| [M+H]+1 \| 146.0349 \| 147.04222 \| -12.84 \| Full match \| \| 35 \| Cryptochlorogenic acid \| C16 H18 O9 \| 4.749 \| [M+H]+1 \| 354.09056 \| 355.09796 \| -12.76 \| Full match \| \| 36 \| Cytosine \| C4 H5 N3 O \| 1.253 \| [M+H]+1 \| 111.04197 \| 112.04952 \| -11.67 \| Full match \| \| 37 \| Dehydroandrographolide \| C20 H28 O4 \| 13.436 \| [M+H]+1 \| 332.19218 \| 333.19946 \| -19.82 \| Full match \| \| 38 \| Demethyleneberberine \| C19 H17 N O4 \| 4.75 \| [M+H]+1 \| 323.11162 \| 324.1189 \| -12.8 \| Full match \| \| 39 \| Desoxyrhaponticin \| C21 H24 O8 \| 5.365 \| [M-H]-1 \| 404.14702 \| 403.13974 \| -0.25 \| Full match \| \| 40 \| Dihydrolycorine \| C16 H19 N O4 \| 11.212 \| [M+H]+1 \| 289.12773 \| 290.13501 \| -12.71 \| Full match \| \| 41 \| Ellagic acid \| C14 H6 O8 \| 5.662 \| [M+H]+1 \| 302.00312 \| 303.00983 \| -10.4 \| Full match \| \| 42 \| Epigoitrin \| C5 H7 N O S \| 0.249 \| [M+H]+1 \| 129.02481 \| 130.03209 \| -0.17 \| Full match \| \| 43 \| Esculetin \| C9 H6 O4 \| 5.039 \| [M-H]-1 \| 178.02521 \| 177.01793 \| -7.88 \| Full match \| \| 44 \| Esculin \| C15 H16 O9 \| 4.765 \| [M-H]-1 \| 340.07897 \| 339.07181 \| -1.37 \| Full match \| \| 45 \| Ethyl 4-methoxycinnamate \| C12 H14 O3 \| 5.162 \| [M+H]+1 \| 206.0921 \| 207.09938 \| -10.63 \| Full match \| \| 46 \| Ethylparaben \| C9 H10 O3 \| 3.765 \| [M+H]+1 \| 166.06074 \| 167.0683 \| -13.6 \| Full match \| \| 47 \| Ferulic acid \| C10 H10 O4 \| 5.302 \| [M-H]-1 \| 194.05667 \| 193.04939 \| -6.38 \| Full match \| \| 48 \| Forsythoside E \| C20 H30 O12 \| 5.265 \| [M-H]-1 \| 462.17344 \| 461.16617 \| -0.61 \| Full match \| \| 49 \| Fraxetin \| C10 H8 O5 \| 4.408 \| [M+H]+1 \| 208.0341 \| 209.04179 \| -14.77 \| Full match \| \| 50 \| Fumaric acid \| C4 H4 O4 \| 1.589 \| [M-H]-1 \| 116.00884 \| 115.0019 \| -18.3 \| Full match \| \| 51 \| Galangin \| C15 H10 O5 \| 6.132 \| [M-H]-1 \| 270.05244 \| 269.04517 \| -1.4 \| Full match \| \| 52 \| Gallic acid \| C7 H6 O5 \| 2.186 \| [M-H]-1 \| 170.01984 \| 169.01253 \| -9.89 \| Full match \| \| 53 \| Gentiopicrin \| C16 H20 O9 \| 5.147 \| [M-H]-1 \| 356.11055 \| 355.1033 \| -0.51 \| Full match \| \| 54 \| Hematoxylin \| C16 H14 O6 \| 5.245 \| [M-H]-1 \| 302.07902 \| 301.07175 \| -0.05 \| Full match \| \| 55 \| Hesperetin \| C16 H14 O6 \| 5.494 \| [M-H]-1 \| 302.07912 \| 301.07184 \| 0.25 \| Full match \| \| 56 \| Higenamine \| C16 H17 N O3 \| 4.728 \| [M-H]-1 \| 271.12059 \| 270.11337 \| -0.93 \| Full match \| \| 57 \| Hydroxygenkwanin \| C16 H12 O6 \| 6.115 \| [M-H]-1 \| 300.06291 \| 299.05563 \| -1.59 \| Full match \| \| 58 \| Hyperoside \| C21 H20 O12 \| 5.313 \| [M-H]-1 \| 464.09337 \| 463.0878 \| -4.54 \| Full match \| \| 59 \| Indigo \| C16 H10 N2 O2 \| 13.4 \| [M+H]+1 \| 262.07101 \| 263.07829 \| -12.27 \| Full match \| \| 60 \| Isobutyl 4-hydroxybenzoate \| C11 H14 O3 \| 13.473 \| [M+H]+1 \| 194.09229 \| 195.09957 \| -10.31 \| Full match \| \| 61 \| Isoguanosine \| C10 H13 N5 O5 \| 1.814 \| [M-H]-1 \| 283.09132 \| 282.08405 \| -1.22 \| Full match \| \| 62 \| Isopropyl 4-Hydroxybenzoate \| C10 H12 O3 \| 4.681 \| [M+H]+1 \| 180.07609 \| 181.08354 \| -14.2 \| Full match \| \| 63 \| Isoscopoletin \| C10 H8 O4 \| 4.936 \| [M+FA-H]-1 \| 192.04136 \| 237.03944 \| -4.67 \| Full match \| \| 64 \| Jatrorrhizine \| C20 H19 N O4 \| 5.269 \| [M+H]+1 \| 337.127 \| 338.13428 \| -13.07 \| Full match \| \| 65 \| L(-)-Carnitine \| C7 H15 N O3 \| 1.201 \| [M+H]+1 \| 161.10331 \| 162.11052 \| -11.71 \| Full match \| \| 66 \| Lactose \| C12 H22 O11 \| 1.508 \| [M+FA-H]-1 \| 342.1158 \| 387.11407 \| -1.2 \| Full match \| \| 67 \| Limonin \| C26 H30 O8 \| 12.861 \| [M+H]+1 \| 470.18832 \| 471.19577 \| -12.21 \| Full match \| \| 68 \| L-Leucine \| C6 H13 N O2 \| 2.31 \| [M+H]+1 \| 131.09312 \| 132.10042 \| -11.53 \| Full match \| \| 69 \| L-Phenylalanine \| C9 H11 N O2 \| 3.027 \| [M+H]+1 \| 165.07703 \| 166.0843 \| -11.82 \| Full match \| \| 70 \| L-Tyrosine \| C9 H11 N O3 \| 1.619 \| [M+H]+1 \| 181.0718 \| 182.07903 \| -11.54 \| Full match \| \| 71 \| L-Valine \| C5 H11 N O2 \| 1.349 \| [M+H]+1 \| 117.07784 \| 118.08511 \| -9.75 \| Full match \| \| 72 \| Maleic acid \| C4 H4 O4 \| 1.918 \| [M-H]-1 \| 116.00924 \| 115.00196 \| -14.84 \| Full match \| \| 73 \| Mannitol \| C6 H14 O6 \| 1.956 \| [M-H]-1 \| 182.07767 \| 181.07039 \| -7.54 \| Full match \| \| 74 \| Methyl 4-hydroxy-3-methoxycinnamate \| C11 H12 O4 \| 4.442 \| [M+H]+1 \| 208.07091 \| 209.07819 \| -12.73 \| Full match \| \| 75 \| Methyl vanillate \| C9 H10 O4 \| 0.37 \| [M-H]-1 \| 182.05522 \| 181.04951 \| -14.74 \| Full match \| \| 76 \| Nepodin \| C13 H12 O3 \| 4.593 \| [M-H]-1 \| 216.08174 \| 215.07446 \| 14.33 \| Full match \| \| 77 \| Orcinol gentiobioside \| C19 H28 O12 \| 4.9 \| [M-H]-1 \| 448.15795 \| 447.1503 \| -0.28 \| Full match \| \| 78 \| Orsellinic acid \| C8 H8 O4 \| 5.334 \| [M-H]-1 \| 168.04405 \| 167.03677 \| 10.66 \| Full match \| \| 79 \| Oxoglaucine \| C20 H17 N O5 \| 11.388 \| [M+H]+1 \| 351.1064 \| 352.11368 \| -12.16 \| Full match \| \| 80 \| Oxyberberine \| C20 H17 N O5 \| 6.291 \| [M+H]+1 \| 351.10669 \| 352.11397 \| -11.35 \| Full match \| \| 81 \| Oxyresveratrol \| C14 H12 O4 \| 2.354 \| [M-H+HAc]-1 \| 244.06907 \| 303.08304 \| -18.38 \| Full match \| \| 82 \| Paeonol \| C9 H10 O3 \| 1.272 \| [M+H]+1 \| 166.06093 \| 167.06821 \| -12.43 \| Full match \| \| 83 \| Palmatine \| C21 H21 N O4 \| 5.713 \| [M+H]+1 \| 351.14266 \| 352.14993 \| -12.54 \| Full match \| \| 84 \| p-Coumaric acid \| C9 H8 O3 \| 4.946 \| [M+H]+1 \| 164.04541 \| 165.05269 \| -11.78 \| Full match \| \| 85 \| p-Hydroxybenzaldehyde \| C7 H6 O2 \| 0.467 \| [M-H]-1 \| 122.03499 \| 121.02772 \| -14.63 \| Full match \| \| 86 \| Poncirin \| C28 H34 O14 \| 5.444 \| [M-H]-1 \| 594.19429 \| 593.18701 \| -0.95 \| Full match \| \| 87 \| Propylparaben \| C10 H12 O3 \| 5.927 \| [M-H]-1 \| 180.07774 \| 179.06998 \| -4.99 \| Full match \| \| 88 \| Protocatechualdehyde \| C7 H6 O3 \| 4.879 \| [M-H]-1 \| 138.02995 \| 137.02267 \| -12.63 \| Full match \| \| 89 \| Protocatechuic acid \| C7 H6 O4 \| 4.35 \| [M-H]-1 \| 154.02487 \| 153.01759 \| -11.27 \| Full match \| \| 90 \| Pyrogallol \| C6 H6 O3 \| 2.559 \| [M-H]-1 \| 126.02997 \| 125.02269 \| -13.69 \| Full match \| \| 91 \| Quercetin \| C15 H10 O7 \| 5.598 \| [M-H]-1 \| 302.04207 \| 301.03494 \| -1.91 \| Full match \| \| 92 \| Quinic acid \| C7 H12 O6 \| 1.909 \| [M-H]-1 \| 192.0621 \| 191.05476 \| -6.72 \| Full match \| \| 93 \| Rotundine \| C21 H25 N O4 \| 4.203 \| [M+H]+1 \| 355.174 \| 356.18127 \| -12.28 \| Full match \| \| 94 \| Rutin \| C27 H30 O16 \| 5.457 \| [M+H]+1 \| 610.14916 \| 611.15387 \| -6.93 \| Full match \| \| 95 \| Salicylic acid \| C7 H6 O3 \| 4.999 \| [M-H]-1 \| 138.03064 \| 137.02269 \| -7.65 \| Full match \| \| 96 \| Scoparone \| C11 H10 O4 \| 5.074 \| [M+H]+1 \| 206.05553 \| 207.06281 \| -11.55 \| Full match \| \| 97 \| Scopoletin \| C10 H8 O4 \| 4.002 \| [M+H]+1 \| 192.04032 \| 193.04735 \| -10.08 \| Full match \| \| 98 \| Sec-O-Glucosylhamaudol \| C21 H26 O10 \| 4.094 \| [M+H]+1 \| 438.14519 \| 439.15247 \| -16.91 \| Full match \| \| 99 \| Shikimic acid \| C7 H10 O5 \| 1.561 \| [M-H]-1 \| 174.05137 \| 173.04408 \| -8.37 \| Full match \| \| 100 \| Spiculisporic acid \| C17 H28 O6 \| 5.832 \| [M-H]-1 \| 328.18843 \| 327.18115 \| -0.49 \| Full match \| \| 101 \| Stachydrine \| C7 H13 N O2 \| 1.233 \| [M+H]+1 \| 143.09328 \| 144.10019 \| -9.41 \| Full match \| \| 102 \| Sudan II \| C18 H16 N2 O \| 13.381 \| [M+H]+1 \| 276.12999 \| 277.13727 \| 13.5 \| Full match \| \| 103 \| Synephrine \| C9 H13 N O2 \| 1.336 \| [M+H]+1 \| 167.09262 \| 168.0999 \| -12 \| Full match \| \| 104 \| Tetrahydroxyxanthone \| C13 H8 O6 \| 5.408 \| [M-H]-1 \| 260.03178 \| 259.02451 \| -1.2 \| Full match \| \| 105 \| Trigonelline HCl \| C7 H7 N O2 \| 1.215 \| [M+H]+1 \| 137.04601 \| 138.05331 \| -12.15 \| Full match \| \| 106 \| Uridine \| C9 H12 N2 O6 \| 1.657 \| [M-H]-1 \| 244.06894 \| 243.06163 \| -2.45 \| Full match \| \| 107 \| Vanillin \| C8 H8 O3 \| 0.569 \| [M-H]-1 \| 152.0456 \| 151.03847 \| -11.49 \| Full match \| \| 108 \| Veratric acid \| C9 H10 O4 \| 11.71 \| [M+H]+1 \| 182.05634 \| 183.06361 \| -8.63 \| Full match \| | | | | | | | | | |
| --- | --- | --- | --- | --- | --- | --- | --- | --- | --- | --- | --- | --- | --- | --- | --- | --- | --- | --- | --- | --- | --- | --- | --- | --- | --- | --- | --- | --- | --- | --- | --- | --- | --- | --- | --- | --- | --- | --- | --- | --- | --- | --- | --- | --- | --- | --- | --- | --- | --- | --- | --- | --- | --- | --- | --- | --- | --- | --- | --- | --- | --- | --- | --- | --- | --- | --- | --- | --- | --- | --- | --- | --- | --- | --- | --- | --- | --- | --- | --- | --- | --- | --- | --- | --- | --- | --- | --- | --- | --- | --- | --- | --- | --- | --- | --- | --- | --- | --- | --- | --- | --- | --- | --- | --- | --- | --- | --- | --- | --- | --- | --- | --- | --- | --- | --- | --- | --- | --- | --- | --- | --- | --- | --- | --- | --- | --- | --- | --- | --- | --- | --- | --- | --- | --- | --- | --- | --- | --- | --- | --- | --- | --- | --- | --- | --- | --- | --- | --- | --- | --- | --- | --- | --- | --- | --- | --- | --- | --- | --- | --- | --- | --- | --- | --- | --- | --- | --- | --- | --- | --- | --- | --- | --- | --- | --- | --- | --- | --- | --- | --- | --- | --- | --- | --- | --- | --- | --- | --- | --- | --- | --- | --- | --- | --- | --- | --- | --- | --- | --- | --- | --- | --- | --- | --- | --- | --- | --- | --- | --- | --- | --- | --- | --- | --- | --- | --- | --- | --- | --- | --- | --- | --- | --- | --- | --- | --- | --- | --- | --- | --- | --- | --- | --- | --- | --- | --- | --- | --- | --- | --- | --- | --- | --- | --- | --- | --- | --- | --- | --- | --- | --- | --- | --- | --- | --- | --- | --- | --- | --- | --- | --- | --- | --- | --- | --- | --- | --- | --- | --- | --- | --- | --- | --- | --- | --- | --- | --- | --- | --- | --- | --- | --- | --- | --- | --- | --- | --- | --- | --- | --- | --- | --- | --- | --- | --- | --- | --- | --- | --- | --- | --- | --- | --- | --- | --- | --- | --- | --- | --- | --- | --- | --- | --- | --- | --- | --- | --- | --- | --- | --- | --- | --- | --- | --- | --- | --- | --- | --- | --- | --- | --- | --- | --- | --- | --- | --- | --- | --- | --- | --- | --- | --- | --- | --- | --- | --- | --- | --- | --- | --- | --- | --- | --- | --- | --- | --- | --- | --- | --- | --- | --- | --- | --- | --- | --- | --- | --- | --- | --- | --- | --- | --- | --- | --- | --- | --- | --- | --- | --- | --- | --- | --- | --- | --- | --- | --- | --- | --- | --- | --- | --- | --- | --- | --- | --- | --- | --- | --- | --- | --- | --- | --- | --- | --- | --- | --- | --- | --- | --- | --- | --- | --- | --- | --- | --- | --- | --- | --- | --- | --- | --- | --- | --- | --- | --- | --- | --- | --- | --- | --- | --- | --- | --- | --- | --- | --- | --- | --- | --- | --- | --- | --- | --- | --- | --- | --- | --- | --- | --- | --- | --- | --- | --- | --- | --- | --- | --- | --- | --- | --- | --- | --- | --- | --- | --- | --- | --- | --- | --- | --- | --- | --- | --- | --- | --- | --- | --- | --- | --- | --- | --- | --- | --- | --- | --- | --- | --- | --- | --- | --- | --- | --- | --- | --- | --- | --- | --- | --- | --- | --- | --- | --- | --- | --- | --- | --- | --- | --- | --- | --- | --- | --- | --- | --- | --- | --- | --- | --- | --- | --- | --- | --- | --- | --- | --- | --- | --- | --- | --- | --- | --- | --- | --- | --- | --- | --- | --- | --- | --- | --- | --- | --- | --- | --- | --- | --- | --- | --- | --- | --- | --- | --- | --- | --- | --- | --- | --- | --- | --- | --- | --- | --- | --- | --- | --- | --- | --- | --- | --- | --- | --- | --- | --- | --- | --- | --- | --- | --- | --- | --- | --- | --- | --- | --- | --- | --- | --- | --- | --- | --- | --- | --- | --- | --- | --- | --- | --- | --- | --- | --- | --- | --- | --- | --- | --- | --- | --- | --- | --- | --- | --- | --- | --- | --- | --- | --- | --- | --- | --- | --- | --- | --- | --- | --- | --- | --- | --- | --- | --- | --- | --- | --- | --- | --- | --- | --- | --- | --- | --- | --- | --- | --- | --- | --- | --- | --- | --- | --- | --- | --- | --- | --- | --- | --- | --- | --- | --- | --- | --- | --- | --- | --- | --- | --- | --- | --- | --- | --- | --- | --- | --- | --- | --- | --- | --- | --- | --- | --- | --- | --- | --- | --- | --- | --- | --- | --- | --- | --- | --- | --- | --- | --- | --- | --- | --- | --- | --- | --- | --- | --- | --- | --- | --- | --- | --- | --- | --- | --- | --- | --- | --- | --- | --- | --- | --- | --- | --- | --- | --- | --- | --- | --- | --- | --- | --- | --- | --- | --- | --- | --- | --- | --- | --- | --- | --- | --- | --- | --- | --- | --- | --- | --- | --- | --- | --- | --- | --- | --- | --- | --- | --- | --- | --- | --- | --- | --- | --- | --- | --- | --- | --- | --- | --- | --- | --- | --- | --- | --- | --- | --- | --- | --- | --- | --- | --- | --- | --- | --- | --- | --- | --- | --- | --- | --- | --- | --- | --- | --- | --- | --- | --- | --- | --- | --- | --- | --- | --- | --- | --- | --- | --- | --- | --- | --- | --- | --- | --- | --- | --- | --- | --- | --- | --- | --- | --- | --- | --- | --- | --- | --- | --- | --- | --- | --- | --- | --- | --- | --- | --- | --- | --- | --- | --- | --- | --- | --- | --- | --- | --- | --- | --- | --- | --- | --- | --- | --- | --- | --- | --- | --- | --- | --- | --- | --- | --- | --- | --- | --- | --- | --- | --- | --- | --- | --- | --- | --- | --- | --- | --- | --- | --- | --- | --- | --- | --- | --- | --- | --- | --- | --- | --- | --- | --- | --- | --- | --- | --- | --- | --- | --- | --- | --- | --- | --- | --- | --- | --- | --- | --- | --- | --- | --- | --- | --- | --- | --- | --- | --- | --- | --- | --- | --- | --- | --- | --- | --- | --- | --- | --- | --- | --- | --- | --- | --- | --- | --- | --- | --- | --- | --- | --- | --- | --- | --- | --- | --- | --- | --- | --- | --- | --- | --- | --- | --- | --- | --- | --- | --- | --- | --- | --- | --- | --- | --- | --- | --- | --- | --- | --- | --- | --- | --- | --- | --- | --- | --- | --- | --- | --- | --- | --- | --- | --- | --- | --- | --- | --- | --- | --- | --- | --- | --- | --- | --- | --- | --- | --- | --- | --- | --- |
| **Supplementary Table6.** Differential metabolites | | | | | | | | | |
| **Metabolite** | **Mode** | **M/Z** | **ZLD-H vs DSS** | |  | **DSS vs Control** | |  | |
|  |  |  | **VIP** | **FC** | **Regulate** | **VIP** | **FC** | **Regulate** | |
| Indicine | positive | 338.138273 | 5.524 | 1.97E-10 | ↑** | 1.7235 | 0.5214 | ↓* | |
| Absinthin | negative | 541.2847392 | 4.2238 | 2.0517 | ↑** | 2.9054 | 0.4905 | ↓*** | |
| 3-(naphthalen-1-ylmethyl)-1h-pyrazolo[3,4-d] pyrimidin-4-Amine | positive | 276.126289 | 3.6493 | 0.000009623 | ↑** | 2.9394 | 0.4734 | ↓*** | |
| Leontogenin | negative | 427.2846939 | 3.4995 | 0.6416 | ↓** | 3.91 | 4.8857 | ↑*** | |
| Homocysteinesulfinic acid | negative | 212.0232919 | 3.3684 | 1.492 | ↑** | 2.0992 | 0.6732 | ↓*** | |
| Deuteroporphyrin | positive | 555.1969664 | 3.0348 | 0.0002873 | ↑* | 2.3703 | 0.7025 | ↓** | |
| Macromomycin B | negative | 294.0624285 | 2.9833 | 1.3507 | ↑*** | 1.584 | 0.7926 | ↓** | |
| Sulfolithocholylglycine | positive | 546.3088727 | 2.881 | 0.00001845 | ↑** | 2.5135 | 0.7349 | ↓*** | |
| Chlorpromazine sulfoxide | negative | 713.1824763 | 2.8635 | 1.2975 | ↑** | 1.9302 | 0.7616 | ↓** | |
| L-Hexahydro-3-imino-1,2,4-oxadiazepine-3-carboxylic acid | negative | 317.124524 | 2.7798 | 1.2804 | ↑** | 1.8127 | 0.7802 | ↓** | |
| Cinncassiol C1 19-glucoside | negative | 577.2073405 | 2.6648 | 1.3041 | ↑* | 1.9578 | 0.7477 | ↓* | |
| Docosahexaenoic Acid | negative | 327.2330663 | 2.6372 | 0.6237 | ↓* | 2.5493 | 2.4467 | ↑** | |
| 6-Ketoestriol | positive | 325.1389896 | 2.5616 | 0.00005941 | ↑** | 2.2135 | 0.7763 | ↓*** | |
| L-cis-Cyclo(aspartylphenylalanyl) | negative | 297.0651446 | 2.5118 | 1.334 | ↑** | 2.626 | 0.617 | ↓*** | |
| Silux | positive | 495.2412349 | 2.4226 | 0.001768 | ↑* | 1.6739 | 0.7838 | ↓* | |
| 9S-hydroxy-11,15-dioxo-5Z,13E-prostadienoic acid | negative | 349.2023472 | 2.3714 | 0.8035 | ↓** | 1.6676 | 1.3057 | ↑** | |
| Zeranol | negative | 303.1620625 | 2.3464 | 1.2175 | ↑** | 1.0986 | 0.8592 | ↓* | |
| 5(S)-HETE | negative | 319.2281204 | 2.3049 | 0.8381 | ↓** | 1.2943 | 1.1241 | ↑** | |
| Docebenone | negative | 347.1635562 | 2.2758 | 1.4323 | ↑** | 1.6473 | 0.7175 | ↓** | |
| 6-Keto-prostaglandin F1a | negative | 351.2212379 | 2.2656 | 1.2406 | ↑* | 2.0214 | 0.7567 | ↓** | |
| 4Alpha-hydroxymethyl-5alpha-cholesta-8,24-dien-3beta-ol | negative | 459.348682 | 2.2281 | 0.8628 | ↓** | 2.0811 | 1.2762 | ↑*** | |
| Stearaldehyde | positive | 307.2417684 | 2.2099 | 0.0006772 | ↑* | 1.1167 | 0.9 | ↓* | |
| Cinobufotalin | negative | 975.4830452 | 2.1985 | 1.2219 | ↑* | 2.177 | 0.716 | ↓** | |
| Butyric acid | negative | 263.1502366 | 2.1908 | 1.2154 | ↑** | 1.1914 | 0.8651 | ↓* | |
| (+)-Dehydrovomifoliol | negative | 443.2426929 | 2.1863 | 0.7516 | ↓* | 1.6818 | 1.5562 | ↑* | |
| Chembl4569322 | negative | 577.2075298 | 2.1444 | 1.2497 | ↑** | 1.9848 | 0.7234 | ↓*** | |
| Quinocarmycin analog | negative | 392.1388419 | 2.1153 | 1.2092 | ↑** | 1.8615 | 0.7627 | ↓*** | |
| Calcifediol lactone | negative | 427.2848068 | 2.1064 | 0.873 | ↓** | 2.188 | 1.3458 | ↑** | |
| Taurocholic acid 3-sulfate | negative | 296.6173054 | 2.0904 | 1.1897 | ↑** | 1.1191 | 0.8876 | ↓* | |
| Hydroxybuprenorphine | negative | 504.2711483 | 1.9284 | 0.8409 | ↓** | 1.1992 | 1.1465 | ↑* | |
| Nordeoxycholic acid | negative | 399.2531134 | 1.8865 | 0.9102 | ↓** | 2.325 | 1.3714 | ↑*** | |
| 7,10,13,16,19-Docosapentaenoic acid | negative | 375.2544261 | 1.8451 | 0.9076 | ↓** | 2.1921 | 1.3373 | ↑*** | |
| Kaurenoic acid methyl ester | negative | 361.2387579 | 1.8145 | 0.9095 | ↓** | 1.6056 | 1.16 | ↑*** | |
| Tetracosapentaenoic acid (24:5n-3) | negative | 403.2851633 | 1.7967 | 0.9198 | ↓** | 1.7553 | 1.1768 | ↑*** | |
| Physagulin F | negative | 543.2636194 | 1.7689 | 1.0739 | ↑** | 1.2269 | 0.9255 | ↓*** | |
| (11S,12S,13S)-Epoxy-hydroxyoctadeca-cis-9-cis-15-dien-1-oic acid | negative | 331.1919278 | 1.7659 | 0.8985 | ↓* | 1.0266 | 1.1023 | ↑* | |
| 12-O-D-Glucuronoside-13-hydroxyoctadec-9Z-enoate | negative | 525.2514536 | 1.7592 | 1.1214 | ↑** | 1.1407 | 0.9014 | ↓** | |
| Allopregnanolone | negative | 363.2544384 | 1.7555 | 0.9145 | ↓* | 2.0489 | 1.2753 | ↑*** | |
| 11-beta-Hydroxyandrosterone-3-glucuronide | negative | 527.2503324 | 1.7397 | 0.8582 | ↓* | 1.2628 | 1.1979 | ↑* | |
| Prostaglandin F3a | negative | 333.2075341 | 1.7386 | 0.9049 | ↓** | 1.3241 | 1.1284 | ↑** | |
| Trans-2-trans-4-Heptadien-1-ol | negative | 157.0864422 | 1.7358 | 1.132 | ↑** | 1.2283 | 0.8734 | ↓** | |
| Bufotalin | negative | 443.2427318 | 1.7185 | 0.8842 | ↓* | 2.114 | 1.3597 | ↑*** | |
| MG (0:0/20:4(5Z,8Z,11Z,14Z)/0:0) | negative | 377.2702939 | 1.7131 | 0.9266 | ↓** | 1.9343 | 1.2106 | ↑*** | |
| Demissidine | negative | 445.332899 | 1.6634 | 0.9305 | ↓** | 1.6018 | 1.1357 | ↑*** | |
| Beta-Tocopherol | negative | 461.3644235 | 1.6318 | 0.8936 | ↓* | 1.5025 | 1.1802 | ↑** | |
| Ganaxolone | negative | 377.2702575 | 1.598 | 0.9191 | ↓* | 1.9109 | 1.2763 | ↑*** | |
| 5alpha-Pregnan-20alpha-ol-3-one | negative | 363.254391 | 1.5685 | 0.9341 | ↓** | 1.8205 | 1.196 | ↑*** | |
| (25R)-3beta,4beta-dihydroxycholest-5-en-26-oate (1-) | negative | 431.3171444 | 1.5668 | 0.9364 | ↓** | 1.6197 | 1.1477 | ↑ | |
| Liquiritigenin | negative | 255.0665126 | 1.5443 | 1.1269 | ↑* | 1.053 | 0.8683 | ↓* | |
| 5b-Dihydrotestosterone | negative | 335.2231753 | 1.5259 | 0.9405 | ↓*** | 1.3021 | 1.0955 | ↑*** | |
| 15(S)-HpEDE | negative | 339.2543055 | 1.5249 | 0.9288 | ↓* | 1.6205 | 1.1734 | ↑*** | |
| Bolasterone | negative | 361.2389289 | 1.5122 | 0.9331 | ↓** | 1.2835 | 1.1006 | ↑*** | |
| Adrenic acid | negative | 377.2701495 | 1.478 | 0.9431 | ↓* | 1.8971 | 1.1969 | ↑*** | |
| Drostanolone | negative | 349.2386676 | 1.4412 | 0.944 | ↓** | 1.7427 | 1.1673 | ↑*** | |
| Arabinosylhypoxanthine | negative | 303.0504833 | 1.44 | 0.9399 | ↓** | 1.3435 | 1.1077 | ↑** | |
| 11-Hydroxyhexadecanoylcarnitine | negative | 450.2993873 | 1.4374 | 0.9393 | ↓** | 1.373 | 1.1201 | ↑*** | |
| Estradiol | negative | 317.1761904 | 1.4281 | 0.9239 | ↓* | 1.2384 | 1.1282 | ↑** | |
| Oryzalide B | negative | 365.197087 | 1.4264 | 0.9351 | ↓* | 1.272 | 1.1166 | ↑** | |
| Methyltestosterone | negative | 347.2232184 | 1.4129 | 0.9398 | ↓** | 1.2109 | 1.0939 | ↑*** | |
| 5-(10,13-Nonadecadienyl)-1,3-benzenediol | negative | 417.3015626 | 1.4118 | 0.9269 | ↓* | 1.7267 | 1.2347 | ↑*** | |
| 11'-Carboxy-gamma-chromanol | negative | 403.2854087 | 1.4112 | 0.9492 | ↓** | 1.4996 | 1.1222 | ↑*** | |
| 5-Hydroxyvalproic acid | negative | 205.1079338 | 1.4013 | 1.0975 | ↑** | 1.0221 | 0.9171 | ↓** | |
| Ubiquinone-2 | negative | 363.1816522 | 1.3817 | 0.9307 | ↓* | 1.1867 | 1.1232 | ↑* | |
| Crustecdysone | negative | 479.3017397 | 1.3768 | 0.9377 | ↓** | 1.3042 | 1.1059 | ↑*** | |
| Docosapentaenoic acid (22n-3) | negative | 375.254636 | 1.3714 | 0.949 | ↓** | 1.4822 | 1.1251 | ↑*** | |
| Gibberellin A37 | positive | 385.1440409 | 1.3665 | 0.00149 | ↑* | 1.7084 | 0.8715 | ↓*** | |
| LysoPC(17:0/0:0) | negative | 508.3404232 | 1.3646 | 0.9259 | ↓** | 1.0321 | 1.0861 | ↑* | |
| LysoPE(P-18:0/0:0) | negative | 464.3150352 | 1.3608 | 0.9283 | ↓** | 1.2748 | 1.1186 | ↑** | |
| DG (18:3(10,12,15)-OH (9)/0:0/8:0) | negative | 475.343915 | 1.3568 | 0.95 | ↓** | 1.709 | 1.1558 | ↑*** | |
| Sterculic acid | negative | 339.2543262 | 1.3532 | 0.9431 | ↓* | 1.5176 | 1.1434 | ↑*** | |
| Bufadienolide | negative | 399.2537526 | 1.3451 | 0.9543 | ↓** | 1.6412 | 1.1434 | ↑*** | |
| Tetrahydrodeoxycorticosterone | negative | 379.2494644 | 1.336 | 0.9417 | ↓* | 1.3056 | 1.1003 | ↑*** | |
| (22R,23R)-22,23-dihydroxy-campest-4-en-3-one | negative | 475.3430836 | 1.3286 | 0.9411 | ↓* | 1.4995 | 1.1482 | ↑*** | |
| MG (14:1(9Z)/0:0/0:0) | negative | 345.2285598 | 1.3002 | 0.9573 | ↓* | 1.113 | 1.07 | ↑** | |
| Polyporusterone E | negative | 481.2971801 | 1.2927 | 0.9506 | ↓** | 1.3039 | 1.0956 | ↑*** | |
| Ecdysone | negative | 509.3114526 | 1.2896 | 0.9382 | ↓* | 1.2587 | 1.1119 | ↑** | |
| 2,6-Dimethylocta-3,7-diene-1,6-diol | negative | 169.1229139 | 1.2799 | 1.1328 | ↑* | 1.8941 | 0.759 | ↓*** | |
| (x)-2-Heptanol glucoside | negative | 259.1552836 | 1.2614 | 1.0664 | ↑** | 1.0395 | 0.9259 | ↓** | |
| Fasoracetam | negative | 241.1192608 | 1.2479 | 0.9376 | ↓* | 1.0207 | 1.1078 | ↑* | |
| 9,10-Dihydroxystearic acid | negative | 315.2541916 | 1.2467 | 0.9541 | ↓* | 1.0334 | 1.0687 | ↑** | |
| Lithocholic acid | negative | 421.2961879 | 1.2282 | 0.9458 | ↓* | 1.6347 | 1.1978 | ↑*** | |
| Prolyl-Valine | negative | 213.1243614 | 1.2184 | 1.0583 | ↑* | 1.0388 | 0.9185 | ↓** | |
| Pregnanetriolone | negative | 349.2386753 | 1.2164 | 0.9583 | ↓* | 1.6322 | 1.1395 | ↑*** | |
| Arachidonic acid | negative | 303.2332217 | 1.2132 | 0.9185 | ↓* | 1.1344 | 1.1211 | ↑* | |
| Propofol | negative | 401.2693955 | 1.2128 | 0.9484 | ↓* | 1.5918 | 1.1636 | ↑*** | |
| Sphingosine 1-phosphate | negative | 424.2472 | 1.1954 | 1.0705 | ↑* | 1.1342 | 0.901 | ↓** | |
| 2-[N-(ethoxymethyl)-2-Ethyl-6-methylanilino]-2-Oxoethanesulfonic Acid | negative | 314.1071469 | 1.166 | 1.0787 | ↑* | 1.1273 | 0.9071 | ↓** | |
| Lovastatin acid | negative | 421.2601382 | 1.1597 | 0.96 | ↓* | 1.2575 | 1.0921 | ↑*** | |
| Contignasterol | negative | 507.3325685 | 1.1425 | 0.9625 | ↓* | 1.1 | 1.0709 | ↑*** | |
| 2-Hydroxydecanedioic acid | negative | 217.1080272 | 1.1334 | 1.0448 | ↑** | 1.1011 | 0.9385 | ↓*** | |
| TetraHCA | negative | 465.3220351 | 1.1096 | 0.9553 | ↓* | 1.2535 | 1.0914 | ↑*** | |
| MG (0:0/20:5(5Z,8Z,11Z,14Z,17Z)/0:0) | negative | 421.2600704 | 1.0967 | 0.9656 | ↓* | 1.1073 | 1.0696 | ↑*** | |
| N-Docosahexaenoyl Tryptophan | negative | 535.297579 | 1.0585 | 0.9534 | ↓** | 1.018 | 1.0682 | ↑** | |
| 3a,17a-Dihydroxy-5b-androstane | negative | 337.2388436 | 1.0578 | 0.9554 | ↓* | 1.0459 | 1.0757 | ↑*** | |
| 3alpha,7alpha,12alpha-trihydroxy-5beta-cholestanate | negative | 449.3277915 | 1.0098 | 0.9689 | ↓* | 1.2755 | 1.0911 | ↑*** | |
| 9-Oxo-nonanoic acid | negative | 217.10803 | 1.0018 | 1.0423 | ↑** | 1.0024 | 0.9414 | ↓*** | |

↑: represents an increase in the expression of metabolites, ↓: represents a decrease in the expression of metabolites; **P* <0.05, ***P* <0.01, ****P* <0.001.
